# Supplementary material for: Digital technology to facilitate Proactive Assessment of Obesity Risk during Infancy (ProAsk): a feasibility study
Source: BMJ Open. 2017 Sep 6;7(9):e017694. doi: 10.1136/bmjopen-2017-017694 (PMC5588959; doi:10.1136/bmjopen-2017-017694)
Supplement: Supplementary file 1 [file bmjopen-2017-017694supp001.pdf]

SUPPLEMENTARY FILE 1 - SUMMARY OF DATA COLLECTION MEASURES

**Methodological issue and associated study objective**	**Quantitative measures**	**Qualitative data collected**
1. Did the feasibility study allow a sample size calculation for the main trial?	Researchers recorded the number of infants recruited.<br>Parent self-report of infant weight and age at baseline and 6 months enabled changes in weight for age z-scores for in infants at average population risk and above population risk of overweight to be calculated.	N/A
2. What factors influenced eligibility and what proportion of those approached were eligible?	The NHS Child Health Records were examined to determine the number of infants that received their 6-8 week check in the respective study sites during the recruitment period.<br>Health visitor (HV) log sheets recorded the number of 6-8 week old infants receiving home visits, the number of parents who were eligible for the study and were approached about participating, the number of parents who declined and their reasons (where given) for declining.	Interviews with HVs to explore practitioners' experiences of recruiting to the feasibility study.
3. Was recruitment successful?	Comparison of expected and observed recruitment.<br>Recruitment rate anticipated to be N=100 in three months (based on data provided by NIHR-CRN co-ordinator). Any extension to the recruitment period.	Interviews with HVs to explore their perspective on the recruitment processes.

4. Did eligible participants consent?	HV records of numbers of eligible infants and numbers of parents who agreed to be approached by the researcher. Researcher records of parents successfully recruited.	Interviews with HVs to explore their perspective on the recruitment processes.
5. Were participants successfully randomised and did randomisation yield equality in groups?	Not assessed	Not assessed
6. Were blinding procedures adequate?	Not assessed	Not assessed
7. Did participants adhere to the intervention?	Proportion of participants who received the ProAsk assessment and intervention for those at greater risk.	Interviews with HVs explored intervention adherence. Cross referencing of data on overweight risk downloaded from the ProAsk tablets with interview data from parents allowed researchers to investigate key elements of intervention adherence.
8. Was the intervention acceptable to the participants?	Numbers of parents and HVs who took part in the study	Interviews with HVs and parents explored their views on the acceptability of the intervention.
9. Was it possible to calculate intervention costs and duration?	Not assessed	Not assessed
10. Were outcome assessments completed	Completion rates for risk assessment, baseline and 6 month follow-up questionnaires.	
11. Were outcomes measured those that were the most appropriate outcomes?	Proportion and type of missing data in ProAsk risk assessment and follow-up measures	Interviews with parents exploring their views of study processes.
12. Was retention to study good?	Retention rate - from consent to follow-up at 6 months.	
13. Were the logistics of running a multicentre trial assessed?	Recruitment and intervention completion rates for all four sites.	Interviews with HVs probed their views of the study processes.

14. Did all components of the intervention work together?	Completion rates of ProAsk assessment.	Interviews with HVs and parents explored the interviewees' experiences of intervention components, including IROC assessment, risk feedback, therapeutic wheel, motivational interviewing techniques, goal setting and follow-up.
