# Supplementary material for: Digital technology to facilitate Proactive Assessment of Obesity Risk during Infancy (ProAsk): a feasibility study
Source: BMJ Open. 2017 Sep 6;7(9):e017694. doi: 10.1136/bmjopen-2017-017694 (PMC5588959; doi:10.1136/bmjopen-2017-017694)
Supplement: Author's manuscript [file bmjopen-2017-017694.draft_revisions.pdf]

BMJ Open

BMJ Open is committed to open peer review. As part of this commitment we make the peer review history of every article we publish publicly available.

When an article is published we post the peer reviewers' comments and the authors' responses online. We also post the versions of the paper that were used during peer review. These are the versions that the peer review comments apply to.

The versions of the paper that follow are the versions that were submitted during the peer review process. They are not the versions of record or the final published versions. They should not be cited or distributed as the published version of this manuscript.

BMJ Open is an open access journal and the full, final, typeset and author-corrected version of record of the manuscript is available on our site with no access controls, subscription charges or pay-per-view fees (http://bmjopen.bmj.com).

If you have any questions on BMJ Open's open peer review process please email info.bmjopen@bmj.com

BMJ Open

DIGITAL TECHNOLOGY TO FACILITATE PROACTIVE ASSESSMENT OF OBESITY RISK DURING INFANCY (PROASK): A FEASIBILITY STUDY

Journal:	*BMJ Open*
Manuscript ID	bmjopen-2017-017694
Article Type:	Research
Date Submitted by the Author:	10-May-2017

Complete List of Authors:	Redsell, Sarah; Anglia Ruskin University, Faculty of Health, Social Care & Education<br>Rose, Jennie; Anglia Ruskin University, Faculty of Health, Social Care & Education<br>Weng, Stephen; University of Nottingham, NIHR School of Primary Care Research<br>Ablewhite, Joanne; University of Nottingham<br>Swift, Judy ; University of Nottingham, Division of Nutritional Sciences<br>Siriwardena, Aloysius; University of Lincoln, School of Health and Social Care<br>Nathan, Dilip; Nottingham University Hospitals NHS Trust<br>Wharrad, Heather; University of Nottingham, School of Health Sciences<br>Atkinson, Pippa; Nottingham City Care Partnership<br>Watson, Vikki; Nottingham City Care Partnership<br>McMaster, Fiona; Anglia Ruskin University, Faculty of Health, Social Care & Education<br>Lakshman, Rajalakshmi; Institute of Metabolic Science, Medical Research Council Epidemiology Unit<br>Glazebrook, Cristine; University of Nottingham, Psychiatry

**Primary Subject Heading**:	Public health
Secondary Subject Heading:	Communication, Paediatrics
Keywords:	Community child health < PAEDIATRICS, PUBLIC HEALTH, PREVENTIVE MEDICINE

SCHOLARONE™ Manuscripts

DIGITAL TECHNOLOGY TO FACILITATE PROACTIVE ASSESSMENT OF OBESITY RISK DURING INFANCY (PROASK): A FEASIBILITY STUDY

TEAM

Professor Sarah Redsell, (SR), Professor of Public Health, Faculty of Health, Social Care & Education, Anglia Ruskin University, East Road Campus, Young Street Site, Cambridge, CB1 1PT. Email: Sarah.Redsell@anglia.ac.uk

Dr Jennie Rose, (JR), Research Fellow, Faculty of Health, Social Care and Education, Anglia Ruskin University. Email: Jennie.Rose@anglia.ac.uk

Dr Stephen Weng, (SW), NIHR Research Fellow, Division of Primary Care, University of Nottingham, Email: Stephen.Weng@nottingham.ac.uk

Dr Joanne Ablewhite, (JA), Research Fellow, Institute of Mental Health, University of Nottingham Innovation Park. Email: joanne.ablewhite1@nottingham.ac.uk

Dr Judy Anne Swift, (JS), Associate Professor of Behavioural Nutrition, Division of Nutritional Sciences, School of Biosciences, University of Nottingham. Email: Judy.Swift@nottingham.ac.uk

Professor A. Niroshan Siriwardena, (ANS), Professor of Primary and Pre-hospital Health Care, Community and Health Research Unit, School of Health and Social Care, University of Lincoln. Email: nsiriwardena@lincoln.ac.uk

Dr Dilip Nathan, (DN), Consultant Paediatrician, Nottingham University Hospitals Trust. Email: Dilip.nathan@nhs.net

Professor Heather Wharrad, (HW), Professor of e-Learning and Health Informatics, School of Health Sciences, University of Nottingham, Email: Heather.Wharrad@nottingham.ac.uk

Mrs Pippa Atkinson, (PA), Lead Health Visitor for Infant Nutrition, Nottingham City Care Partnership. Email: Pippa.Atkinson@nottinghamcitycare.nhs.uk

Mrs Vicki Watson, (VW), Specialist Public Health Dietician, Nottingham City Care Partnership. Email: Vicki.Watson@nottinghamcitycare.nhs.uk

Dr Fiona McMaster, (FM), Senior Lecturer in Public Health, Faculty of Medical Sciences, Anglia Ruskin University Email: Fiona.McMaster@anglia.ac.uk

Dr Rajalakshmi Lakshman, (RL), Consultant in Public Medicine (Lead for Children), Cambridgeshire and Peterborough Public Health Directorate, Shire Hall, Castle Hill, Cambridge, CB3 0AP. Email: Rajalakshmi.Lakshman@mrc-epid.cam.ac.uk

Professor Cris Glazebrook, (CG), Professor of Health Psychology,
Room B12, Institute of Mental Health, University of Nottingham Innovation Park, Triumph Road, Nottingham NG7 2TU. Email: Cris.Glazebrook@nottingham.ac.uk

For peer review only

For peer review only - http://bmjopen.bmj.com/site/about/guidelines.xhtml

ABSTRACT

Objective

To assess the feasibility and acceptability of using digital technology for Proactive Assessment of Obesity Risk during Infancy (ProAsk) with UK Health Visitors (HVs) and parents.

Design

Multi-centre, pre and post intervention feasibility study with process evaluation.

SETTING

Rural and urban deprived settings, UK community care.

Participants

66 parents of infants and 22 HVs.

Intervention

ProAsk was delivered on a tablet device. It comprises a validated risk prediction tool, to quantify overweight risk status, and a therapeutic wheel detailing motivational strategies for preventive parental behaviour. Parents were encouraged to agree goals for behaviour change with HVs who received motivational interviewing training.

Outcome measures

We assessed recruitment, response and attrition rates. Demographic details were collected, including overweight risk status, and the primary outcome measure of weight-for-age z-score, using the WHO growth charts. Qualitative interviews ascertained the acceptability of study processes and intervention fidelity.

Results

HVs screened 324/589 infants for inclusion in the study and 66/226 (29%) eligible infants were recruited. Assessment of overweight risk was completed on 53 infants and 40% of these were identified as above population risk. Weight-for-age z score (SD) between the infants at population risk and those above population risk differed significantly at baseline (-0.67 SD vs 0.32 SD). HVs were able to collect data and calculate overweight risk for the study infants. However, protocol adherence and intervention fidelity was a challenge. HVs and parents found the additional information provided in the therapeutic wheel appropriate and acceptable.

Conclusion

There were significant problems with study recruitment and protocol adherence. ProAsk was acceptable to most parents and HVs but intervention fidelity was low. There was limited evidence to support the feasibility of implementing ProAsk without significant additional resources. A future study could evaluate ProAsk as a stand-alone, parent led-digital intervention or as a HV supported, parent-led intervention.

For peer review only - http://bmjopen.bmj.com/site/about/guidelines.xhtml

STRENGTHS AND LIMITATIONS OF THIS STUDY

This study was the first to examine the feasibility of using the Infant Risk of Overweight Checklist (IROC) prediction algorithm to differentiate between infants at population and above population risk of being overweight.

Qualitative interviews providing both parent and HVs' perspectives on the feasibility of conducting a future RCT and implementing ProAsk within the proposed design were a study strength.

The successful recruitment of some parents from socially deprived areas demonstrated that obesity prevention interventions can be implemented in hard to reach populations.

The main challenges were misinterpretation of participant eligibility by health visitors resulting in less than adequate recruitment and varying levels of intervention fidelity.

ADDITIONAL INFORMATION

TRIAL REGISTRATION NUMBER

NCT02314494

FUNDING

This work was supported by the Medical Research Council – Public Health Intervention Development Scheme, grant number PHIND 01/14-15.

AUTHORS CONTRIBUTION

The authors have no competing interests.

SR, PI for study and lead author, obtained NHS ethics permission, assembled team, accessed gatekeepers, project managed the study, wrote the first draft of the manuscript.

JR responsible for day to day study coordination at two sites, recruited participants, collected and analysed data, contributed to the drafting of the manuscript.

SW responsible for data processing and analysis, preparing quantitative tables and results, editing, and preparation of manuscript drafts.

JA responsible for day to day study coordination at two sites, recruited participants, collected and analysed data, and contributed to a revision of the manuscript.

JS advised on study design, was involved in data analysis and interpretation of findings and contributed to revision of the manuscript.

ANS advised on study design, was involved in interpretation of findings and contributed to a revision of the manuscript.

DN advised on study design, was involved in interpretation of the findings and contributed to a revision of the manuscript.

For peer review only - http://bmjopen.bmj.com/site/about/guidelines.xhtml

HW responsible for the design and development of the ProAsk digital tool, contributed to study design and revision of the manuscript.

PA contributed to the content of the therapeutic wheel and the information that accompanied it. Contributed to the co-ordination of the training for HVs and feedback meetings. Provided local Trust data.

VW contributed to the content of the therapeutic wheel and the information that accompanied it and to revision of the manuscript.

FMc delivered HV training in motivational interviewing, contributed to text within ProAsk digital tool and revision of the manuscript.

RL contributed to study design and revision of the manuscript.

CG contribution to study design, development of ProAsk digital tool, study management and revision of the manuscript.

DATA SHARING STATEMENT

There is unpublished data in relation to the parents' responses to the pre and post questionnaires which can be obtained by contacting the PI. The unpublished qualitative data is being analysed by the authors for a separate publication.

ACKNOWLEDGMENTS

We are grateful to the parents and members of the health visiting teams who took part in this study. We particularly wish to thank Catherine Shiels and Fleur Seekins who managed the health visiting service in the study sites.

For peer review only

For peer review only - http://bmjopen.bmj.com/site/about/guidelines.xhtml

BACKGROUND

Obesity is a global public health challenge that affects all ages. In 2015, over 42 million children under the age of five were overweight [1]. In the UK in 2015/2016, over a fifth (22.1%) of children aged 4-5 years were either overweight or obese [2], with the highest rates in those living in socioeconomically deprived areas [2, 3] or of Asian or Black ethnicity [2]. Although children are born with genetic predispositions related to weight and growth [4], dietary behaviour is modulated by feeding experience and the family environment [5]. Interventions that aim to address these practices have a role in childhood obesity prevention [6].

Parents and health professionals have called for reliable and valid methods of identifying infants at risk of developing childhood overweight and obesity [7-9]. This is possible because the risk factors for childhood overweight and obesity are identifiable antenatally and during infancy [10, 11]. These include parental weight, smoking during pregnancy, birth weight and rapid weight gain; with breastfeeding being moderately protective. Of these, the strongest risk factor for childhood overweight is rapid infant weight gain [12-14]. Between 25%-33% [15, 16] of infants gain weight more rapidly than desirable during the first 6 months of life [17-19] and this risk factor is potentially modifiable if identified during early life. The Infant Risk of Overweight Checklist (IROC) [12, 20], developed using data from the Millennium Cohort Study (MCS), [21] and externally validated using the Avon Longitudinal Study of Parents and Children (ALSPAC) data [22], operationalises an algorithm to predict an infant's future risk of being overweight. IROC [12, 20] offers the opportunity to identify infants at greatest risk of overweight in clinical practice.

Interactive digital technology can support complex and sensitive discussions between health professionals and patients [23-25]. It is also suited to delivering personalised information about health status and risk [26]. Potential behaviour change can be raised through the neutrality of the technological device without increasing anxiety [27]. Proactive Assessment of Obesity Risk during Infancy (ProAsk) is a novel, interactive digital intervention designed to equip Health Visitors (HVs) with an individual infant's risk of future overweight, whilst also supporting discussions with parents. It incorporates the IROC [12, 20] and a therapeutic wheel which has previously been found to be an acceptable format for cancer patients [28]. The therapeutic wheel comprises an interactive graphic detailing strategies for preventive behaviour using supportive and strengths-based prompts. The content of the therapeutic wheel is based on the behavioural strategies identified in a systematic review of interventions that reduce the risk of childhood obesity in early life [6]. ProAsk is designed to be delivered alongside Motivational Interviewing (MI) which has been successfully used to improve health behaviours including diet and physical activity for parent-child dyads [29, 30].

This is the first intervention designed to identify an infant's risk of overweight and intervene with parents of infants at greatest risk to prevent childhood overweight and obesity. There is no evidence for the effectiveness of this approach to draw on to guide our study design, and so, in line with the MRC's framework for the of development and evaluation of complex interventions, [31] a feasibility study was required.

METHOD

STUDY AIM AND OBJECTIVES

For peer review only - http://bmjopen.bmj.com/site/about/guidelines.xhtml

This study aimed to examine the feasibility and acceptability of undertaking a randomised controlled trial (RCT) of ProAsk with UK HVs and parents. We used the ADePT framework [32] to examine the methodological feasibility, and to address the study's objectives which were:

(i) To assess recruitment, response and attrition rates for parents/legal guardians/carers of infants;
(ii) To determine the proportion of infants calculated as at risk using ProAsk at baseline to inform a sample size calculation for a future RCT;
(iii) To evaluate the feasibility of HV delivery of the ProAsk intervention to eligible parents/legal guardians/carers, including an assessment of intervention fidelity and protocol adherence;
(iv) To determine the acceptability and feasibility of the proposed primary and secondary outcomes measures.

Design

Multi-centre, pre and post intervention feasibility study with process evaluation.

Participants and recruitment

ProAsk was delivered by HVs to parents in four study sites in two localities, in urban and rural deprived areas within the UK. One day's training in recruitment processes, ProAsk and MI [33] was delivered to members of the HV teams (N=47). Delivering the intervention required the skills of a HV but to ensure the team understood study processes we trained nursery nurses (N=12), an administrator (N=1), student nurses (N=3), managers (N=3), together with HVs (N=28). The MI training was delivered by a member of the motivational interviewing network of trainers (MINT) and comprised interactive and experiential activities [34] including agenda-mapping and reflective listening. HVs were also offered half a day top-up training in MI during the study period and six HVs from two sites attended. Recruitment commenced on 22nd April 2015 in two sites and 12th June in the other sites, and ended on 30th November 2015.

The local NHS Trusts HV managers estimated that the numbers of births within the study areas over a three month period was approximately 700. The National Institute for Health Research- Clinical Research Network (NIHR-CRN) estimated that HVs could recruit up to 30% of eligible parents to the study. This should provide a pragmatic sample of at least N=100 infants recruited over a three month period.

Parents of infants aged 6-8 weeks were eligible to take part. Exclusion criteria were (a) infant with known medical conditions requiring special diets, (b) mother with diagnosis of post-natal depression (PND) or score of moderate PND or above on health visitor applied screening tools (EPDS >13) [35] (PHQ-9 >10) [36] or anxiety (GAD-7 score >10) [37], (c) infant born before 32 weeks gestation (d) infant birth weight below 2nd centile (e) parent with insufficient English to complete the questionnaires in the absence of face-to-face translation.

Health visitors were asked to recruit all infants attending for their 6-8 week check over a three month period or until the sample size was met. They identified eligible parents at a routine infant check and approached them with information about the study. HVs recorded reasons for exclusion, response to approach, and reasons for refusal on a log sheet. Interested participants gave their permission to be contacted by the researchers by telephone to arrange a home visit, where informed written consent

For peer review only - http://bmjopen.bmj.com/site/about/guidelines.xhtml

was obtained. Where parents did not respond to the initial phone call the researchers made two further calls at different times of the day. At the end of the study a purposively diverse sample of parent participants were invited to take part in qualitative interviews together with all of the HVs.

Figure 1 shows the per protocol study regimen.

INSERT FIGURE 1

ETHICS AND RESEARCH GOVERNANCE PERMISSIONS

Permission to conduct the study was provided by East of England (Essex) NHS Research Ethics Committee on 26th February 2015 (Reference number 15/EE/0011). Research governance permissions were provided by the two NHS Trusts covering the study localities.

INTERVENTION

HVs used a hand-held device (tablet) to deliver ProAsk to parents when their infants were three months old. This involved entering the IROC [12] items (baby birth weight and length, current weight, maternal and paternal height and weight, maternal smoking status during pregnancy, and breastfeeding) into ProAsk which then calculated the infant's risk status using the World Health Organisation (WHO) growth charts [38]. This was displayed on the tablet screen as either "Your baby's risk of being above a healthy weight is the same as other babies" (population risk) or "Your baby's risk of being above a healthy weight is more than other babies" (above population risk). Responses were stored on the password protected tablet. Two tablets were provided per site. Problems with internet access at two sites resulted in an amendment to the data extraction method and HVs were asked to screen shot the IROC result for transfer to the research team.

HVs were asked to offer parents who received the above population risk message an opportunity to explore the therapeutic wheel (Figure 2). This interactive graphic promoted evidence-based behaviour change strategies [39] in four areas: active play; milk and solid foods; sleeping and soothing; and infant feeding cues. It prompted HVs to use a motivational approach [33] to build parental self-efficacy for agreed behaviour goals, which were recorded on leaflets left in the home as cues to action for behavioural change.

INSERT FIGURE 2

MEASURES AND DATA COLLECTION

Demographic details, ethnicity and information about family size were collected at baseline (infant age two months) via a self-report questionnaire completed by parents. Details of the infants IROC score were recorded at three months. Infant anthropometric data, details of infant feeding (breast or formula milk or both) and validated measures of parenting self-efficacy [40, 41] and maternal feeding style (Infant Feeding Questionnaire (IFQ)) [42] were collected by self-report questionnaire at infant age 2 months (baseline) and 6 months (follow-up). In addition, exposure to opportunities for physical activity and sedentary behaviour was recorded by parents at baseline and follow-up as time spent unrestricted on tummy, and restricted in a baby seat, car seat or pushchair.

Parents were interviewed about the acceptability of ProAsk, study processes, including recruitment, and intervention fidelity. HVs were interviewed to explore their experiences of recruiting parents to the study and conducting the ProAsk Assessment. They were also asked about environmental factors

For peer review only - http://bmjopen.bmj.com/site/about/guidelines.xhtml

such as the compatibility of ProAsk with existing workplace goals, organisational barriers and support for the intervention, and their views on the quality of training provided by the team. Interviews lasting up to 90 minutes, were conducted face-to-face and over the telephone, and recorded using a digital Dictaphone.

DATA ANALYSIS

Recruitment, response and attrition rates, demographic details, weight-for-age z score, and overweight risk status (population risk vs. above population risk at 10% risk threshold) were analysed using descriptive statistics via STATA 13 MP4.

Audio data from qualitative interviews with parents and HVs were transcribed verbatim and transcripts were imported into Nvivo software for sorting, coding and categorising. Data relating to relevant methodological issues were subject to thematic content analysis using the method outlined by Boyatzis (1998) [43]. Verbatim quotes illustrate the themes.

RESULTS

The results for each of the ADePT framework's [32] methodological issues are summarised in Table 1. The demographic data are detailed in Table 2.

INSERT TABLES 1 AND 2

The results of the thematic content analysis of the parent (N=12) and HV (N=15) interviews are presented in Tables 3a and 3b.

INSERT TABLES 3A AND 3B

SAMPLE SIZE CALCULATIONS

A total of 324 infants were screened by the HVs during a routine a 6-8 week check, but consent was obtained from only 66 parent-infant dyads (20%). An overweight risk assessment was completed for 56/66 infants and the data transferred to the research team for 53 of these. This showed that 40% infants were above population risk. Sufficient data were collected to inform a sample size calculation but our findings suggest that more attention to study design is needed prior to future evaluation of ProAsk.

ELIGIBILITY

The study flow chart presented in Figure 3 details participant eligibility and the reasons for exclusion. The number of 6-8 week checks logged by the NHS Child Health Records during the extended recruitment phase was 589 which was fewer than the 700 estimated in three months by the NHS Trusts. HVs screened only 324 of these (45%) during the extended recruitment period (3 to 5.5 months for one locality and seven for the other).

INSERT FIGURE 3

In the HV interviews, language was identified as a major barrier to participant eligibility (N=9), particularly in one site (Table 2a, N=7). HVs (N=8) were also concerned about referring parents with mental health, safeguarding or domestic violence issues.

For peer review only - http://bmjopen.bmj.com/site/about/guidelines.xhtml

> ***HV N20:*** *It was the language barrier really. I'd say one hundred percent or ninety nine percent of my parents are non-English speaking so obviously without an interpreter.*

> ***HV C37:*** *Because there were other issues around perhaps, safeguarding, in need, other agencies working with that family, and yet something else for them to have to deal with.*

Recruitment

The recruitment target of N-100 infants in three months was not met. The most common reasons for parents declining were: parents not interested (N=28) and parents lacked time (N= 21). The sample contained more than the expected number of mothers with degree level education. The Income Deprivation Affecting Children Index (IDACI) [44], which measures area deprivation based on post-code, for participants with completed risk assessment showed that more (33%) of the participants recruited were from the two lower quintiles than from the two upper quintiles (25%) (Table 2).

In total, 22/28 HVs who received training took part in the study (the remaining HVs were transferred, elsewhere, or on sickness or maternity leave). Most HVs interviewed took part at the request of their managers. Workload was identified as a barrier to parent recruitment by 5/15 HVs interviewed. Six reported being wary of raising the study with parents (Table 3a).

Of the 12 parents interviewed, 11 found the study recruitment processes acceptable and 10 felt well-informed. Seven parents participated because of concerns about their own weight and a further seven did so for altruistic reasons. Eleven parents were willing to be randomised for a future trial around identification and intervention with infants at future risk of overweight.

Consent

Of the 138 parents who gave permission to be contacted by the researchers, 66 (48%) provided written consent (Figure 3). Interviews with HVs suggested that one reason for the low conversion rate to written consent was that parents were wary of accepting a telephone call or arranging a home visit with unknown researchers.

> ***HV N9:*** *So I think that then when I said someone else would come in after me, some families were not keen to take part. Half our battle is for us to get in, then when I said someone else, I found that was hard.*

Adherence to intervention

In total, 56/66 infants had their overweight risk score calculated. Interviews with the HVs suggested that the main reason that ten parents did not receive their risk assessment was that they were not at home when the HV came to deliver ProAsk.

> ***HV C22:*** *A couple of them I've been to their house at the designated time and they haven't been there so I haven't revisited them. Because you know, if you go out and see them and they're not there what do you do? You perhaps have to chase them up but to honest I haven't had the time.*

An important element was for HVs to feedback the overweight risk score to parents, but four parents were not aware of having received this feedback or were uncertain as to what it meant for them. Four HVs interviewed reported difficulties feeding back to parents the overweight risk score.

For peer review only - http://bmjopen.bmj.com/site/about/guidelines.xhtml

Although goal-setting and follow-up contact was recommended for infants identified as being above population risk of overweight, this did not always take place. Goal-setting around behaviour change was recorded for only five of the 21 parents whose infants were at above population risk. HV interviews confirmed that of the 11 HVs who had conducted a ProAsk assessment and were interviewed, seven had shown parents all elements of the wheel rather than focussing on one specific area. Three of the HVs interviewed had used the therapeutic wheel to provide information to all participants, irrespective of their risk score status.

> ***HV C22:*** *I know when I did the actual wheel, if you like, you said to discuss one topic, we ended up discussing them all. Because all of those topics are covered in health visiting anyway, to me it didn't feel right that we talked about diet without exercise and feeding cues.*

Intervention acceptability

A total of 88 parents declined participation. Eight out of 12 parent participants found ProAsk acceptable and were positive about its digital functionality. One parent expressed disappointment with ProAsk There was evidence that ProAsk helped to engage parents and avoided information overload.

> ***Parent C8:*** *I thought the information in there was really nice and visual actually, because sometimes you can hear a lot of information and it's sort of difficult to absorb it and there was quite a lot of it, it was nice to have something in front of you as well as you were having that discussion. A sort of a visual prompt you could refer back to.*

Six out of 28 HVs did not take part in the study. Eight HVs expressed initial concerns about the unintended consequences of communicating overweight risk status, particularly at infant age 3 months which some considered too early for personalised risk communication.

> ***HV N5****: I mean when I first heard about the research I was quite concerned initially because I had visions of mothers sort of starving their babies that's more of a risk than over feeding a baby at that sort of very early age, delicate age when they're so young.*

However, HVs also recognised the potential benefits of early intervention to prevent overweight with seven stating they found the therapeutic wheel engaging to use and containing useful information. One HV suggested that the intervention was disappointing.

Outcome assessment

Table 2 shows the demographic and participant characteristics at baseline stratified by overweight risk status. At the 10% risk threshold, 32 (60%) infants were at average population risk and 21 (40%) were above. There was a statistically significant difference in birth weight (3.28 vs 3.86) and weight-for-age z score (-0.67 vs 0.32) between the infants that were at population risk and those above population risk at baseline. There was also a significant difference in paternal BMI (26.5 vs 30.1kg/m^2) but not maternal BMI or smoking status.

Selection of outcomes

The parent self-report measures were completed fully by 85% of respondents. However, there was missing data for infant length and head circumference because these measures were no longer routinely recorded in parent-held infant records. Cronbach's alpha for the parenting self-efficacy [40,

For peer review only - http://bmjopen.bmj.com/site/about/guidelines.xhtml

41] and maternal feeding style (Infant Feeding Questionnaire (IFQ)) [42] measures all exceeded >0.5, indicating acceptable internal consistency [45, 46].

At interview, four parents described how completing the baseline questionnaire had prompted them to change their behaviour around infant opportunities for active play and sedentariness. This was an unexpected measurement effect.

> ***Parent C16:*** *When I filled in the questionnaire, at least the first time I filled them in, there were a few that made me think about how I could change it. For example there was a question about how much tummy time the baby gets. And I'd never really thought of that as a form of exercise, which I then started to do. And it made me more aware of trying to get my son that tummy time. It made me think it's not all about what they eat, it's about, well exercising the calories off.*

Study retention

In total, 34/66 parents returned the follow-up questionnaire at six months (51% retention rate). Three of these had not received the intervention. All parents (N=12) invited to participate in post-study interviews agreed to take part.

Logistics of multicentre trial

One site recruited more parents than the others (see Table 1). HVs from this site were able to overcome teething problems through team working and reaching out to the researcher for support. The HVs from the other sites talked about the teams or their own resistance to the study because they felt their geographical area was unsuitable for the study.

> ***HV C43:*** *Our administrator was brilliant; I knew you were on the end of the phone; I had support from my peers, if we didn't know how to do something we worked it out between us.*

> ***HV N20.*** *It was just said that the area has been chosen so that was fine. I just think that it was the wrong area, absolutely totally the wrong area.*

Components of protocol working together

The low level of fidelity suggests there were incompatibilities between the risk assessment and preventative strategies within ProAsk. HVs found it difficult to find the time for additional home visits to complete the behaviour change aspect of the intervention.

> ***HV: C22*** *I have to say we are a bit stretched for time, we're short of staff, and it is another visit that we have to fit in on top of everything else. So from that point of view, it was a bit stressful I suppose.*

Discussion

The aim of the study was to determine the feasibility and acceptability of conducting an RCT of ProAsk. Parents and HVs found the study processes acceptable and ProAsk engaging. However, HVs had reservations about assessing and communicating overweight risk to parents of young infants. Overall recruitment to the study was lower than expected. Poor conversion of potential participants to consent resulted in the study failing to meet the recruitment target. There were problems with

For peer review only - http://bmjopen.bmj.com/site/about/guidelines.xhtml

protocol adherence and intervention fidelity, with some parents not receiving all elements of the intervention.

This study was conducted in areas identified as being socially deprived because childhood obesity is more prevalent [2, 3]. Whilst recruitment was disappointing, the IDACI [44] scores for the infant participants show that more socioeconomically deprived households were included. The fact that 40% of infants recruited were assessed as being at risk of overweight further demonstrates that our target group was sampled. Recruiting participants from these areas is known to be challenging [47]. Other research studies have used opt-in [48] or financial incentives [49] to improve recruitment in socially disadvantaged areas. However, the potential for parental stigmatisation [50] make such strategies less applicable to studies of overweight prevention. Language was a significant barrier to recruitment, particularly at one site where most of the parents did not speak English as their first language. Future research will need to ensure that interpreting and translation service are resourced.

The sample contained a relatively high proportion of participants with degree level education. HV logs of 6-8 week visits and interviews with HVs indicated that some groups of parents such as those with a history of mental health concerns, were not approached about the study, even if they were eligible for participation, because of concerns about their ability to deal with study burden. There is evidence from other settings that professional gatekeepers do not approach all participants eligible for healthcare research [51-54]. In this study 45% of potentially eligible infants were not screened and a future study should employ dedicated researchers to screen and recruit all potential participants.

HVs were wary about risk assessment and some had anxieties about raising the topic of weight with parents. Ten infants did not receive the risk assessment which was explained in terms of parents being unavailable but may also reflect HVs hesitancy. Problems with the technology in the field resulted in the data from three infants not transferring from tablet devices to the research team. Most HVs adopted a universal approach to intervention showing parents all the preventative information available on the therapeutic wheel rather than guiding them towards their own goals for behaviour change. HV service schedule advises them to use a motivational approach [55] but it may be that the risk assessment conflicted with the goal-setting aspect of ProAsk, and they were unable to switch between approaches. The additional training we provided may not have enabled HVs to transfer their existing skills in MI to the study requirements. The next phase of the study will need to consider whether HVs need more bespoke training and support to deliver the behaviour change components of the intervention.

Parents found ProAsk engaging and the use of digital technology acceptable. Other studies have shown similar acceptability and engagement with digital interventions resulting in improved retention of information and advice that had not been retained following a consultation [56, 57]. The ProAsk therapeutic wheel could be adapted to provide accessible digital information for parents and carers which would address their request for on-going information in digital format.

The researchers were able to collect data on outcomes of interest from parents at the times specified in the protocol. A minority of parents reported that the questionnaire items around infant activity and sedentary behaviour led them to consider behaviour change which may be a contamination risk for a future RCT. Respondent agreement between the validated outcome measures varied from poor to good, with higher levels of internal consistency in the follow up questionnaire.

For peer review only - http://bmjopen.bmj.com/site/about/guidelines.xhtml

CONCLUSIONS

The study identified significant problems with study recruitment and protocol adherence. Many of these problems could be addressed by employing dedicated researchers to screen and recruit participants. Although the intervention was acceptable to most parents and HVs interviewed, the fidelity of delivery was disappointing. There was limited evidence to support the feasibility of adding ProAsk to HV's role without significant additional resources. A future study could evaluate ProAsk as a stand-alone, parent led-digital intervention or as a HV supported, parent-led intervention.

REFERENCES

1. **UNICEF, WHO, The World Bank: Joint Child Malnutrition Estimates - Levels and Trends: Key findings of the 2016 edition**
2. NHS Digital: **National Child Measurement Programme**. In.; 2015/2016.
3. Shrewsbury V, Wardle, J: **Socioeconomic status and adiposity in childhood: a systematic review of cross-sectional studies 1990-2005**. *Obesity* 2008, **16**(2):275-284.
4. Elks CE, Loos RJ, Hardy R, Wills AK, Wong A, Wareham NJ, Kuh D, Ong KK: **Adult obesity susceptibility variants are associated with greater childhood weight gain and a faster tempo of growth: the 1946 British Birth Cohort Study**. *Am J Clin Nutr* 2012, **95**(5):1150-1156.
5. Dubois L, Ohm, KK, Girard, M, Tatone-Tokuda, F, Pérusse, D, Hjelmborg, J, Skytthe, A, Rasmussen, F, Wright, MJ, Lichtenstein, P, Martin, NG: **Genetic and Environmental Contributions to Weight, Height, and BMI from Birth to 19 Years of Age: An International Study of Over 12,000 Twin Pairs**. *PLoS ONE* 2012, **7**(2):e30153.
6. Redsell S, Edmonds B, Nathan D, Siriwardena AN, Swift J, Weng SF, Glazebrook C: **Systematic review of behavioural and non-behavioural interventions, delivered during infancy and the antenatal period, that reduce the risk of overweight and obesity in childhood** *Matern Child Nutr* 2015, **April** (DOI: 10.1111/mcn.12184).
7. Redsell S, Atkinson PJ, Nathan D, Siriwardena AN, Swift JA, Glazebrook C: **Parents' beliefs about appropriate infant size, growth and feeding behaviour: implications for the prevention of childhood obesity**. *BMC Public Health* 2010, **10**(1):711.
8. Redsell S, Atkinson PJ, Nathan D, Siriwardena AN, Swift JA, Glazebrook C: **Preventing childhood obesity during infancy in UK primary care: a mixed-methods study of HCPs' knowledge, beliefs and practice** *BMC Family Practice* 2011, **12**:54.
9. Redsell S, Swift JA, Nathan D, Siriwardena AN, Atkinson P, Glazebrook C: **UK health visitors' role in identifying and intervening with infants at risk of developing obesity**. *Matern Child Nutr* 2013, **9**(3):396-408.
10. Weng SF, Redsell SA, Swift JA, Yang M, Glazebrook CP: **Systematic review and meta-analyses of risk factors for childhood overweight identifiable during infancy**. *Archives of Disease in Childhood* 2012, **97**(12):1019-1026.
11. Woo Baidal JA, Locks LM, Cheng ER, Blake-Lamb TL, Perkins ME, Taveras EM: **Risk Factors for Childhood Obesity in the First 1,000 Days: A Systematic Review: A Systematic Review**. *American Journal of Preventive Medicine* 2016, **50**(6):761-779.
12. Weng S, Redsell, SA, Nathan, D, Swift, JA, Yang, M, Glazebrook, C: **Developing an algorithm to estimate overweight risk in childhood from predictors during infancy**. *Pediatrics,* 2013, **132**(2):e414-e421.
13. Elks CE LR, Sharp SJ, Langenberg C, Ring SM, Timpson NJ, Ness AR, Davey Smith G, Dunger DB, Wareham NJ, Ong KK.: **Genetic markers of adult obesity risk are associated with greater early infancy weight gain and growth**. *PLoS Med* 2010, **7**(5):e1000284.
14. Druet C, Stettler N, Sharp S, Simmons RK, Cooper C, Smith GD, Ekelund U, Levy-Marchal C, Jarvelin MR, Kuh D, Ong KK: **Prediction of childhood obesity by infancy weight gain: an individual-level meta-analysis**. *Paediatric and Perinatal Epidemiology* 2012, **26**(1):19-26.
15. Ekelund U, Ong K, Linne Y, Neovius M, Brage S, Dunger DB, Wareham NJ, Rossner S: **Upward weight percentile crossing in infancy and early childhood independently predicts fat mass**

For peer review only - http://bmjopen.bmj.com/site/about/guidelines.xhtml

in young adults: the Stockholm Weight Development Study (SWEDES). *American Journal of Clinical Nutrition* 2006, **83**(2):324-330.

16. Hui L, Schooling CM, Leung SS, Mak KH, Ho LM, Lam TH, Leung GM: **Birth weight, infant growth, and childhood body mass index: Hong Kong's children of 1997 birth cohort**. *Archives of Pediatrics & Adolescent Medicine* 2008, **162**(3):212-218.
17. Ong K, Loos R: **Rapid infancy weight gain and subsequent obesity: systematic reviews and hopeful suggestions**. *Acta Paediatr* 2006, **95**:904 - 908.
18. Baird J, Fisher D, Lucas P, Kleijnen J, Roberts H, Law C: **Being big or growing fast: systematic review of size and growth in infancy and later obesity**. *BMJ* 2005, **331**(7522):929.
19. Monteiro P, Victora CG: **Rapid growth in infancy and childhood and obesity in later life--a systematic review**. *Obes Rev* 2005, **6**(2):143-154.
20. Redsell SA WS, Swift JA, Nathan D, Glazebrook C. : **Validation, optimal threshold determination and clinical utility of the Infant Risk of Overweight Checklist (IROC) for early prevention of child obesity** *Childhood Obesity* 2016, **12**(3):202-209.
21. **Centre for Longitudinal Studies: Millenium Cohort Study**
22. **Avon Longitudinal Study of Parents and Children (ALSPAC)** [http://www.bristol.ac.uk/alspac/]
23. Ferguson M, Brandreth M, Brassington W, Leighton P, Wharrad H: **A Randomized Controlled Trial to Evaluate the Benefits of a Multimedia Educational Program for First-Time Hearing Aid Users**. *Ear & Hearing* 2016, **0**:1-14.
24. Raaff C, Glazebrook C, Wharrad H: **A systematic review of interactive multimedia interventions to promote children's communication with health professionals: implications for communicating with overweight children**. *BMC Medical Informatics and Decision Making* 2014, **14**(1):8-8.
25. Manning JC, Latif A, Carter T, Cooper J, Horsley A, Armstrong M, Wharrad H: **'Our Care through Our Eyes': a mixed-methods, evaluative study of a service-user, co-produced education programme to improve inpatient care of children and young people admitted following self-harm**. *BMJ open* 2015, **5**:e009680-e009680.
26. Widmer RJ, Allison, T. G., Keane, B., Dallas, A., Lerman, L. O., Lerman, A.: **Using an online, personalized program reduces cardiovascular risk factor profiles in a motivated, adherent population of participants**. *American heart journal* 2014, **167**(1):93-100.
27. Glazebrook C, Garrud P, Avery A, Coupland C, Williams H: **Impact of a multimedia intervention "Skinsafe" on patients' knowledge and protective behaviors**. *Preventive Medicine* 2006.
28. Gordon D: **Breaking Barriers: Management of Cancer-related Pain (Educational CD ROM)** *The Clinical Journal of Pain* 2009, **25**(4):351.
29. Borrelli B, Tooley, EM, Schott-Sheldon, LAJ: **Motivational interviewing for parent-child health interventions: a systematic review and meta-analysis**. *Pediatric dentistry* 2016, **37**(3):254-265.
30. Resnicow K, McMaster, F, Bocian, A, Harris, D, Zhou, Y, Snetselaar, L, Schwartz, R, Myers, E, Gotlieb, J, Foster, J, Hollinger, D, Smith, K, Woolford, S, Mueller, D, Wasserman. RC: **Motivational Interviewing and Dietary Counseling for Obesity in Primary Care: An RCT.** *Pediatrics* 2015, **135**(4):649-657.
31. Craig P, Dieppe P, Macintyre S, Michie S, Nazareth I, Petticrew M: **Developing and evaluating complex interventions: the new Medical Research Council guidance**. *BMJ* 2008, **337**.
32. Bugge C, Williams B, Hagen S, Logan J, Glazener C, Pringle S, Sinclair L: **A process for Decision-making after Pilot and feasibility Trials (ADePT): development following a feasibility study of a complex intervention for pelvic organ prolapse.** *Trials* 2013, **14**:353.
33. Miller WR, Rollnick S: **Motivational Interviewing: Helping People Change**, 3 edn; 2013.
34. Edwards E, Stapleton, P, Williams, K, Ball, L: **Building skills, knowledge and confidence in eating and exercise behaviour change: Brief motivational interviewing training for healthcare providers**. *Patient Education and Counseling* 2015, **98**(5):674-676.

For peer review only - http://bmjopen.bmj.com/site/about/guidelines.xhtml

35. Cox J, Holden, JM, Sagovsky, R. : **Detection of postnatal depression: Development of the 10-item Edinburgh Postnatal Depression Scale**. *British Journal of Psychiatry* 1987, **150**(782-786).

36. Spitzer RL KK, Williams JB, et al. : **Validation and utility of a self-report version of PRIME-MD: the PHQ primary care study**. *JAMA* 1999, **282**(19):1737-1744.

37. Spitzer RK, K, Williams, JBW, Löwe, B,: **A brief measure for assessing generalized anxiety disorder: The GAD-7**. *Archives of Internal Medicine* 2006, **166**(10):1092-1097.

38. World Health Organisation WHO: **Child Growth Standards - Weight Velocity**. In.

39. Redsell SA, Edmonds B, Swift JA, Siriwardena AN, Weng S, Nathan D, Glazebrook C: **Systematic review of randomised controlled trials of interventions that aim to reduce the risk, either directly or indirectly, of overweight and obesity in infancy and early childhood**. *Maternal & Child Nutrition* 2016, **12**(1):24-38.

40. Johnston C, Mash, E.J.: **A measure of parenting satisfaction and efficacy**. *Journal of Clinical Child Psychology* 1989, **18**:167-175.

41. Gilmore L, Cuskelly, M.: **Factor structure of the Parenting Sense of Competence Scale using a normative sample**. *Child Care, Health & Development* 2009, **38**:48-55.

42. Baughcum A, Powers, SW, Johnson, SB, Chamberlin, LA, Deeks, CM, Jain, A, Whitaker, RC: **Maternal feeding practices and beliefs and their relationships to overweight in early childhood**. *J Dev Behav Pediatr* 2001, **22**(6):391-408.

43. Boyatzis R (ed.): **Transforming Qualitative Information: Thematic Analysis and Code Development**. Thousand Oaks, California: Sage Publications; 1998.

44. HM Government Department for Communities and Local Government: **Income Deprivation Affecting Children Index (IDACI)**. In.; 2015.

45. Bowling A: **Research Methods in Health: Investigating health and health services**. Buckingham: Open University Press; 2002.

46. Schmitt M: **Uses and abuse of coefficient alpha.** *Psychol Assessment* 1996, **8**:350-353.

47. Emmel N, Hughes K, Greenhalgh J, Sales A: **Accessing socially excluded people - Trust and the gatekeeper in the researcher-participant relationship**. *Sociological Research Online* 2007, **12**(2).

48. Parry O, Bancroft, A, Gnich, W, Amos, A.: **Nobody home? Issues of respondent recruitment in areas of deprivation** *Critical Public Health,* 2011, **11**(4):305-317.

49. Jennings CG, MacDonald TM, Wei L, Brown MJ, McConnachie L, Mackenzie IS: **Does offering an incentive payment improve recruitment to clinical trials and increase the proportion of socially deprived and elderly participants?** *Trials* 2015, **16**:80.

50. Puhl RM, Heuer, C. A.: **Obesity stigma: important considerations for public health**. *American Journal of Public Health* 2010, **100**(6):1019-1028.

51. Mytton J IJ, Manns S, Stevens T, Mulvaney C, Blair P, Powell J, Potter B, Towner E, Emond A, Deave T, Thomas J, Kendrick D, Stewart-Brown S: **The feasibility of using a parenting programme for the prevention of unintentional home injuries in the under-fives: a cluster randomised controlled trial**. *Health Technology Assessment* 2014, **18**(3).

52. Preston NJ FM, Walshe CE, et al.: **Strategies Designed to Help Healthcare Professionals to Recruit Participants to Research Studies**: John Wiley & Sons, Ltd; 2016.

53. Bonevski B, Randell M, Paul C, Chapman K, Twyman L, Bryant J, Brozek I, Hughes C: **Reaching the hard-to-reach: a systematic review of strategies for improving health and medical research with socially disadvantaged groups**. *BMC Med Res Methodol* 2014, **14**:42.

54. Hughes-Morley A, Young B, Waheed W, Small N, Bower P: **Factors affecting recruitment into depression trials: Systematic review, meta-synthesis and conceptual framework**. *Journal of affective disorders* 2015, **172**:274-290.

55. NHS Constitution: **National Health Visiting Service Specification**. In.; 2014/2015.

56. Ferguson M, Brandreth, M., Brassington, W. Wharrad, H.: **Information retention and overload in first-time hearing aid users: an interactive multimedia educational solution.** . *American Journal of Audiology* 2015, **24**:329-322.

For peer review only - http://bmjopen.bmj.com/site/about/guidelines.xhtml

57. Szeszak S MR, Love A, Langmack G, Wharrad H, Dineen RA: **Animated educational video to prepare children for MRI without sedation: evaluation of the appeal and value.** . *Pediatric Radiology* 2016, **46**(12):1744-1750.

For peer review only

Figure 1 Study Regimen

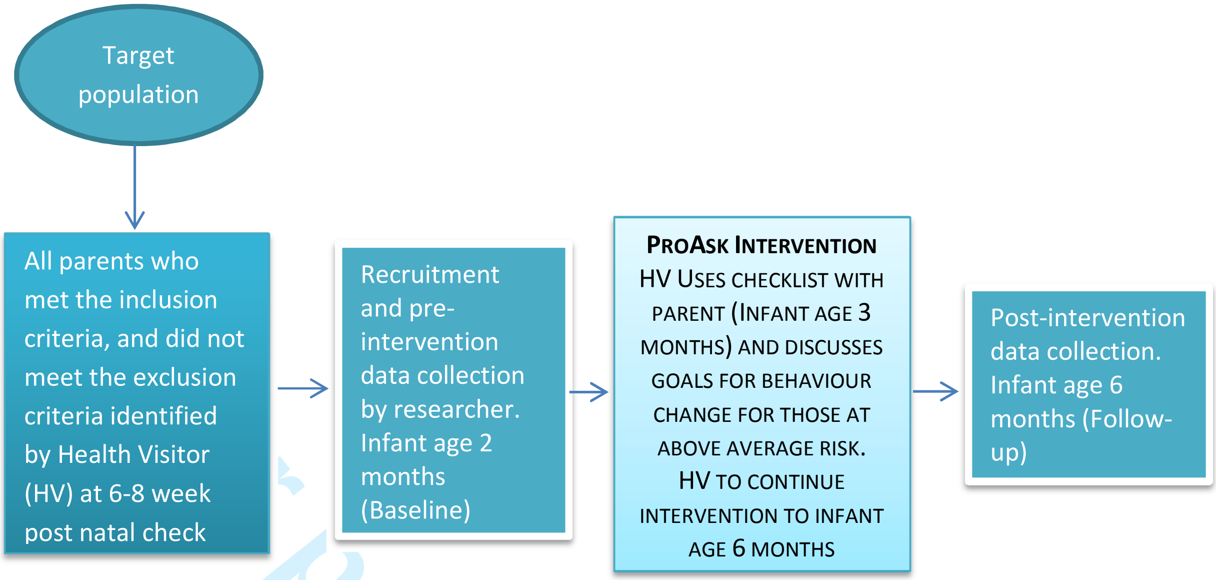

For peer review only - http://bmjopen.bmj.com/site/about/guidelines.xhtml

Figure 2 Therapeutic wheel showing the options to support healthy weight

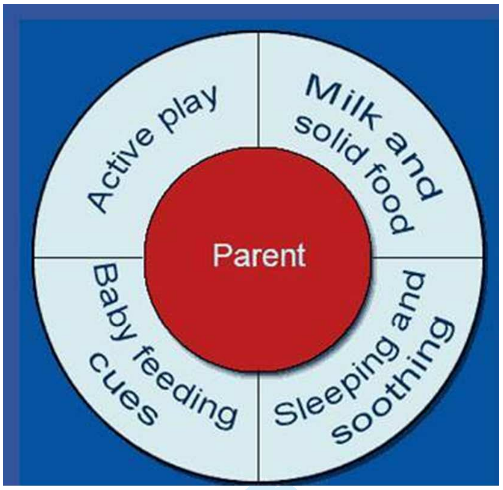

For peer review only

Figure 3 Study Recruitment Flow Chart

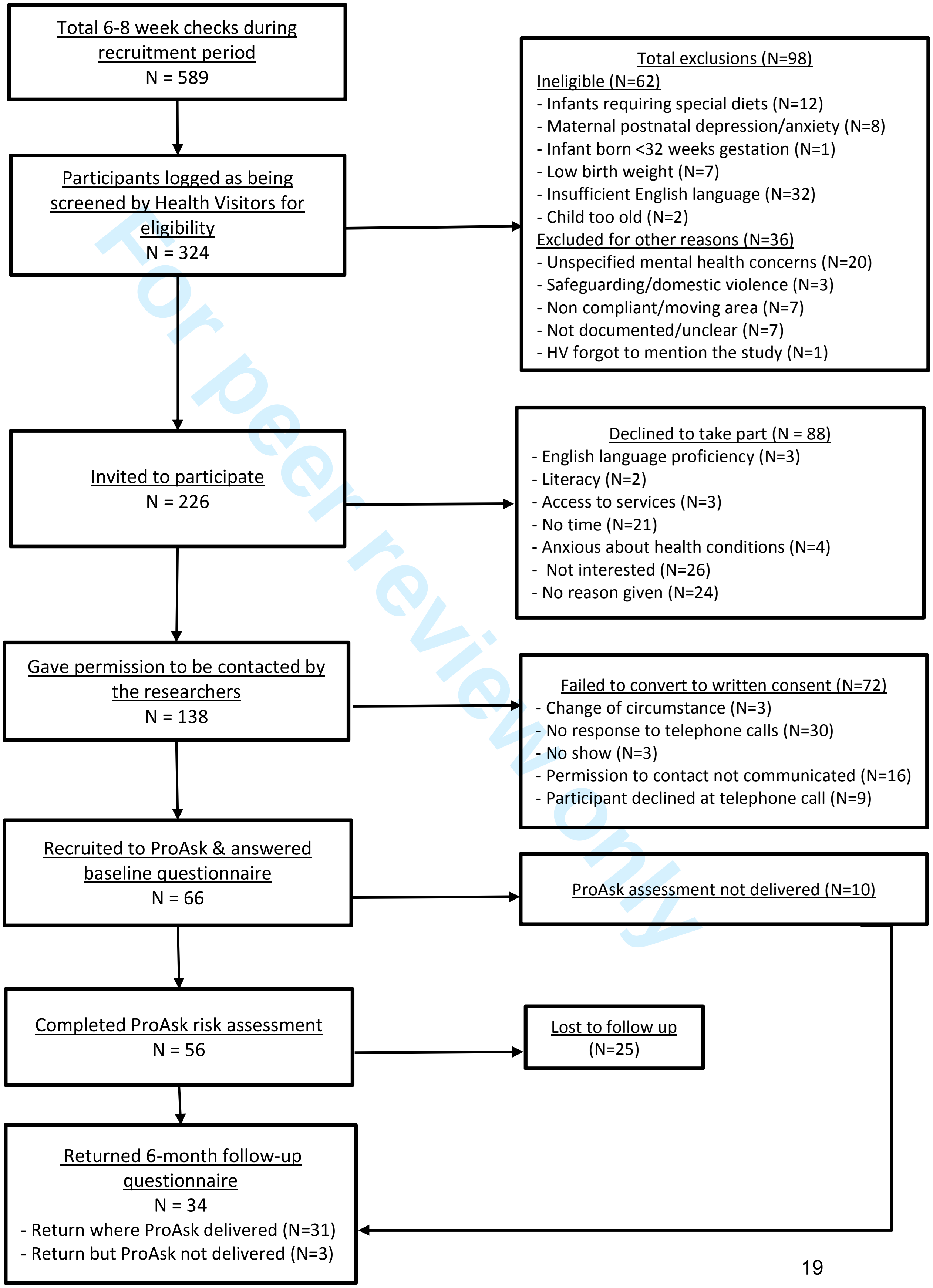

For peer review only - http://bmjopen.bmj.com/site/about/guidelines.xhtml

TABLE 1 (see separate file)

For peer review only

For peer review only - http://bmjopen.bmj.com/site/about/guidelines.xhtml

TABLE 2 DESCRIPTIVE DATA FOR N=53[1] PARTICIPANTS WHO COMPLETED PROASK ASSESSMENT AT BASELINE

Demographic Factors (N=53)	At Population Risk (< 10%)	Above Healthy Risk (≥10%)	**Total**
Gender Boy (%)<br>Girl (%)	15 (46.9)<br>17 (53.1)	12 (57.1)<br>9 (42.9)	**27 (50.8)**<br>**26 (49.1)**
Child age (weeks)	9.84 (1.5)	10.35 (2.3)	**10.04 (1.9)**
Birth weight (kgs)	3.28 (0.5)*	3.86 (0.4)*	**3.51 (0.5)**
Weight for age z-score (WFA-z)	-0.67 (0.6)*	0.32 (0.5)*	**-0.26 (0.7)**
Rapid weight gain (< 0.67 SD)<br>No (%)<br>Yes (%)	 30 (93.8)<br>2 (6.2)	 17 (80.9)<br>4 (19.1)	 **47 (88.7)**<br>**6 (11.3)**
Smoking in pregnancy<br>No (%)<br>Yes (%)	 31 (96.9)<br>1 (3.1)	 21 (100)<br>0 (0)	 **52 (98.1)**<br>**1 (1.9)**
Maternal BMI (kg/m^2)<br>**2 missing values*	24.8 (7.2)	27.7 (8.7)	**25.9 (7.9)**
Paternal BMI (kg/m^2)<br>**15 missing values*	26.5 (4.6)*	30.1 (4.2)*	**28.0 (4.8)**
Feeding choice<br>Exclusive breastfeeding (%)<br>Mixed formula and breast (%)<br>Formula only (%)	 11 (34.4)<br>4 (12.5)<br>17 (53.1)	 11 (52.4)<br>2 (9.5)<br>8 (38.1)	 **22 (41.5)**<br>**6 (11.3)**<br>**25 (47.2)**
Mother marital status<br>Married/Living with Partner (%)<br>Single/Separated (%)	 29 (93.6)<br>2 (6.4)	 21 (100)<br>0 (0)	 **50 (96.2)**<br>**2 (3.8)**
Mother employment status<br>Unemployed (%)<br>Part-time (%)<br>Full-time (%)	 3 (9.4)<br>4 (12.5)<br>25 (78.1)	 4 (19.1)<br>5 (23.8)<br>12 (57.1)	 **7 (13.2)**<br>**9 (17.0)**<br>**37 (69.8)**
Education of mother<br>GCSE (%)<br>A Levels (%)<br>Degree (%)<br>Unknown (%)	 12 (37.5)<br>7 (21.9)<br>13 (40.6)<br>0 (0.0)	 8 (38.1)<br>2 (9.5)<br>9 (42.9)<br>2 (9.5)	 **20 (37.7)**<br>**9 (17.0)**<br>**22 (41.5)**<br>**2 (3.8)**
Number of children<br>One (%)<br>Two (%)<br>Three or more (%)<br>Unknown (%)	 11 (34.4)<br>9 (28.1)<br>5 (15.6)<br>7 (21.8)	 7 (33.3)<br>6 (28.6)<br>5 (23.8)<br>3 (14.3)	 **18 (33.9)**<br>**15 (28.3)**<br>**10 (18.9)**<br>**19 (18.9)**
Ethnicity of child<br>White British (%)<br>Non-White British/Mixed/Other (%)	 28 (87.5)<br>4 (12.5)	 19 (90.5)<br>2 (9.5)	 **47 (88.7)**<br>**6 (11.3)**

For peer review only - http://bmjopen.bmj.com/site/about/guidelines.xhtml

ProAsk Risk Score	5.05 (2.3)*	19.4 (8.7)*	**10.7(9.1)**
Income deprivation affecting children(IDACI) ,2015 (N=56)			
Quintile 1 (%)	3 (9)	5 (24)	**8 (15)**
Quintile 2 (%)	4 (13)	6 (29)	**10 (19)**
Quintile 3 (%)	14 (44)	8 (38)	**22 (42)**
Quintile 4 (%)	8 (25)	2 (10)	**10 (19)**
Quintile 5 (%)	3 (9)	0 (0)	**3 (6)**

1. N=56 infants had a ProAsk assessment but three participants had incomplete data transfer from HV to research team.
Categorical variables are numbers and proportions
Continuous variables are means and standard deviations
$*p < 0.05$

For peer review only

TABLE 3A CONTENT ANALYSIS OF QUALITATIVE DATA FOR HEALTH VISITORS (HVS) (N=15)

	No of participants	No of references
**Eligibility**		
Perception that the study is inappropriate for families where there are maternal mental health concerns or safeguarding issues	8	13
6-8 weeks is a difficult time for HVs to approach parents	8	14
Language barriers problematic	9	18
**Recruitment**		
HV participated at manager's request	9	15
HV workload made it difficult to prioritise study	5	6
Wariness about raising the study with some parents because of expectations that they might be offended or overloaded	6	6
HV engagement with project was supported by positive professional relationships	4	11
Belief that more educated parents were more interested in the study	6	9
**Consent**		
Belief that some parents need a relationship with a professional before they will engage/permit home access	6	11
Belief that parental wariness of unknown researcher negatively impacted on the numbers of participants giving written consent	4	7
**Adherence to intervention**		
Difficulties feeding back overweight risk score from programme to parents	4	8
Therapeutic wheel used with all parents	3	5
All elements of the wheel discussed	7	12
**Intervention Acceptability**		
Concern about unintended consequences of overweight risk identification	8	14
Belief that early prevention is better than later management	9	13
Belief that timing of ProAsk personalised risk communication for infants is too early for parents	9	17
ProAsk is engaging to use	7	18
ProAsk wheel (on tablet) contained useful information	7	9
ProAsk tablet a disappointment	1	1
3-4 months is an appropriate time for this intervention	3	3
**Components of protocol working together**		
Belief that HVs already do this work	7	10
Training day should have been closer to study start date	7	9
Lack of tablet device during training a problem	5	7
Initial lack of confidence in explaining study to potential participants	4	6

For peer review only - http://bmjopen.bmj.com/site/about/guidelines.xhtml

HV misunderstood study protocol	5	6
ProAsk on tablet a challenge for novel users	8	18
Belief that ProAsk risk assessment should be accompanied by intervention and input by HV	6	21
Team work and practice improved skills in using tablet	6	15

For peer review only

For peer review only - http://bmjopen.bmj.com/site/about/guidelines.xhtml

TABLE 3B CONTENT ANALYSIS OF QUALITATIVE DATA FOR PARENTS (N=12)

	No of participants	No of references
**Recruitment**		
Parent felt study recruitment processes acceptable	11	13
Parent felt well informed about the study	10	11
Parent participated because of own weight issues/issues with family eating patterns	7	12
Parent participated for altruistic reasons	7	12
Parent willing to be randomised to participate in future trial.	11	11
**Adherence to intervention**		
No recall of, or uncertainty about, feedback of personalised overweight risk score for baby	4	7
Raised awareness/change in perception in response to overweight risk feedback and intervention	3	3
Parent reports no behaviour change following ProAsk	4	5
**Intervention Acceptability**		
Parental belief that they are already doing the right thing	6	9
Belief that early prevention is better than cure	3	5
Receiving risk score was upsetting	1	1
Receiving risk score was a relief	2	2
Parent would have liked more e.g. app, website, on-going information	3	5
**Outcome assessment**		
Questionnaire components a cue to behaviour change	4	5
**Components of protocol working together**		

For peer review only

For peer review only - http://bmjopen.bmj.com/site/about/guidelines.xhtml

TABLE 1 SUMMARY OF METHODOLOGICAL ISSUES

Methodological issue	Findings	Evidence
1. Did the feasibility study allow a sample size calculation for the main trial?	Response rate less than expected but absolute numbers potentially allow for sample size calculation for main trial once a suitable design has been determined.	See Figure 3. 66/324 (20%) eligible infants were recruited<br>56/66 infants had an IROC assessment, but data from three assessments was missing due to data transfer errors, resulting in 53 complete assessments.<br>40% of infant assessed for overweight risk were above population risk
2. What factors influenced eligibility and what proportion of those approached were eligible?	Fewer infants attending for 6-8 week check in the study areas.<br>High number of screened infants were reported not to have met the inclusion/exclusion criteria (see Figure 3).	HV Managers at the NHS Trusts estimated there would be 700 infants born into the study areas within a three month period. NHS Child Health Records showed 589 estimated potential participants in the study period of which 324 (45%) were identified by HVs. It is not known how many of the infants who were unrecorded by the HVs might have been eligible for the study. HVs recorded screening 324 (55%) parents. Of these, 226 (70%) were recorded as eligible for the study.
3. Was recruitment successful?	Recruitment target not met despite extending the recruitment period extended from 3 to 5 months.<br><br>Challenges at individual and team level	66 parents recruited in 5.5 months and 7 months.<br><br>Not all HVs who were trained took part in the study (22/28). Some did not recruit parents to the study.
4. Did eligible participants consent?	Low conversion to consent.	226 infants were eligible<br>88 (38%) parents refused to participate.<br>138 parents were identified as being interested in

For peer review only

		the study. A failure of communication between researchers and some HVs meant that 16 parents who had given their permission to be contacted were not followed up. Researchers were unable to get responses to their telephone calls from 22% (30/138) of parents who initially expressed interest in the study.<br>66 (48%) consented to participate.
5. Were participants successfully randomised and did randomisation yield equality in groups?	Not assessed	
6. Were blinding procedures adequate?	Not assessed	
7. Did participants adhere to the intervention?	Not all parents had a ProAsk assessment/viewed the therapeutic wheel.	56/66 infants received the risk assessment. Interviews with HVs revealed that all 10 parents who did not receive an assessment were not at home when they called. 21/53 were found to be at above population risk but only five of these received the therapeutic wheel intervention per protocol.<br>Process evaluation revealed that N=4 parents received the assessment but did not receive the risk score feedback.
8. Was the intervention acceptable to the participants?	Some parents and HVs didn't take part in the study.<br><br>Low number of parents consenting suggest some were concerned about the intervention	6/28 HVs declined to take part in the study. Qualitative interviews suggests they were wary of raising issue of infant weight but adjusted their practice to incorporate ProAsk.<br>88 (38%) parents refused to take part following initial interest. Parent participants found the intervention acceptable.

For peer review only

9. Was it possible to calculate intervention costs and duration?	Not assessed	
10. Were outcome assessments completed	HVs were able to complete risk score on 56 participants.<br><br>Outcome measures collected appropriate data Follow-up questionnaire completion rate low.	See Table 2 for more details of outcome data.<br><br>34/66 parents completed follow-up questionnaire, of which 29 were usable.
11. Were outcomes measured those that were the most appropriate outcomes?	Missing anthropometric data mainly in relation to infant length and head circumference at both baseline and follow-up.<br><br>Low numbers of parents recorded the date of the anthropometric measures on the questionnaires. Of those that did there was a date discrepancy between completion of the self-report aspect of the questionnaires (by parent at home) and the associated anthropometric measures (collected in clinic).<br>Parents completed secondary outcome measures consistently.	Number of anthropometric measures completed<br>*Baseline (total N=66)*<br>Weight N=64, Length N=26, Head circumference N=34.<br>*Follow-up (total N=34)*<br>Weight N=28, Length N=15, Head circumference N=14.<br>The anthropometric measures on the baseline questionnaire that were dated (N=13) were recorded on average 22 days earlier than the parent report data. The anthropometric measures on the follow-up questionnaire that were dated (N=8) were recorded on average 15 days earlier.<br><br>Cronbach's alpha for all three measures was >0.5
12. Was retention to study good?	Retention was low.	34/66 (52%) of parents completed follow-up questionnaire. The average time to complete was 28 weeks (min 23, max 39 weeks).<br>Because the intervention was not delivered per protocol there was a time lapse between the

For peer review only

		assessment and follow-up.<br>12 parents agreed to post-study qualitative interviews.
13. Were the logistics of running a multicentre trial assessed?	One centre recruited better than the others. This team work well together and there were low levels of staff change and conflict.	Site 1 recruited N=39/109 eligible infants (36%),<br>Site 2 N=15/83 (18%),<br>Site 3 N= 3/10 (30%),<br>Site 4 N= 9/38 (24%).
14. Did all components of the intervention work together?	There were some difficulties blending the ProAsk risk assessment and the motivational approach.<br><br>The Motivational Interviewing (MI) training was too early and insufficient for some HVs.	ProAsk delivered in its entirety to most parents. Only five HVs completed the goal setting stage resulting in a failure to offer follow-up care to parents of infants identified as at above average risk of overweight.<br>Mismatch between MI training and implementation timing.

For peer review only

SUPPLEMENTARY FILE 1 - SUMMARY OF DATA COLLECTION MEASURES

**Methodological issue and associated study objective**	**Quantitative measures**	**Qualitative data collected**
1. Did the feasibility study allow a sample size calculation for the main trial?	Researchers recorded the number of infants recruited.<br>Parent self-report of infant weight and age at baseline and 6 months enabled changes in weight for age z-scores for in infants at average population risk and above population risk of overweight to be calculated.	N/A
2. What factors influenced eligibility and what proportion of those approached were eligible?	The NHS Child Health Records were examined to determine the number of infants that received their 6-8 week check in the respective study sites during the recruitment period.<br>Health visitor (HV) log sheets recorded the number of 6-8 week old infants receiving home visits, the number of parents who were eligible for the study and were approached about participating, the number of parents who declined and their reasons (where given) for declining.	Interviews with HVs to explore practitioners' experiences of recruiting to the feasibility study.
3. Was recruitment successful?	Comparison of expected and observed recruitment.<br>Recruitment rate anticipated to be N=100 in three months (based on data provided by NIHR-CRN co-ordinator). Any extension to the recruitment period.	Interviews with HVs to explore their perspective on the recruitment processes.
4. Did eligible participants consent?	HV records of numbers of eligible infants and	Interviews with HVs to explore their

For peer review only

	numbers of parents who agreed to be approached by the researcher. Researcher records of parents successfully recruited.	perspective on the recruitment processes.
5. Were participants successfully randomised and did randomisation yield equality in groups?	Not assessed	Not assessed
6. Were blinding procedures adequate?	Not assessed	Not assessed
7. Did participants adhere to the intervention?	Proportion of participants who received the ProAsk assessment and intervention for those at greater risk.	Interviews with HVs explored intervention adherence. Cross referencing of data on overweight risk downloaded from the ProAsk tablets with interview data from parents allowed researchers to investigate key elements of intervention adherence.
8. Was the intervention acceptable to the participants?	Numbers of parents and HVs who took part in the study	Interviews with HVs and parents explored their views on the acceptability of the intervention.
9. Was it possible to calculate intervention costs and duration?	Not assessed	Not assessed
10. Were outcome assessments completed	Completion rates for risk assessment, baseline and 6 month follow-up questionnaires.	
11. Were outcomes measured those that were the most appropriate outcomes?	Proportion and type of missing data in ProAsk risk assessment and follow-up measures	Interviews with parents exploring their views of study processes.
12. Was retention to study good?	Retention rate - from consent to follow-up at 6 months.	
13. Were the logistics of running a multicentre trial assessed?	Recruitment and intervention completion rates for all four sites.	Interviews with HVs probed their views of the study processes.
14. Did all components of the intervention work together?	Completion rates of ProAsk assessment.	Interviews with HVs and parents explored the interviewees' experiences of intervention

For peer review only

		components, including IROC assessment, risk feedback, therapeutic wheel, motivational interviewing techniques, goal setting and follow-up.

For peer review only

DIGITAL TECHNOLOGY TO FACILITATE PROACTIVE ASSESSMENT OF OBESITY RISK DURING INFANCY (PROASK): A FEASIBILITY STUDY

Journal:	*BMJ Open*
Manuscript ID	bmjopen-2017-017694.R1
Article Type:	Research
Date Submitted by the Author:	07-Jul-2017

Complete List of Authors:	Redsell, Sarah; Anglia Ruskin University, Faculty of Health, Social Care & Education<br>Rose, Jennie; Anglia Ruskin University, Faculty of Health, Social Care & Education<br>Weng, Stephen; University of Nottingham, NIHR School of Primary Care Research<br>Ablewhite, Joanne; University of Nottingham<br>Swift, Judy ; University of Nottingham, Division of Nutritional Sciences<br>Siriwardena, Aloysius; University of Lincoln, School of Health and Social Care<br>Nathan, Dilip; Nottingham University Hospitals NHS Trust<br>Wharrad, Heather; University of Nottingham, School of Health Sciences<br>Atkinson, Pippa; Nottingham City Care Partnership<br>Watson, Vikki; Nottingham City Care Partnership<br>McMaster, Fiona; Anglia Ruskin University, Faculty of Health, Social Care & Education<br>Lakshman, Rajalakshmi; Institute of Metabolic Science, Medical Research Council Epidemiology Unit<br>Glazebrook, Cristine; University of Nottingham, Psychiatry

**Primary Subject Heading**:	Public health
Secondary Subject Heading:	Communication, Paediatrics
Keywords:	Community child health < PAEDIATRICS, PUBLIC HEALTH, PREVENTIVE MEDICINE

For peer review only - http://bmjopen.bmj.com/site/about/guidelines.xhtml

DIGITAL TECHNOLOGY TO FACILITATE PROACTIVE ASSESSMENT OF OBESITY RISK DURING INFANCY (PROASK): A FEASIBILITY STUDY

TEAM

Professor Sarah Redsell, (SR), Professor of Public Health, Faculty of Health, Social Care & Education, Anglia Ruskin University, East Road Campus, Young Street Site, Cambridge, CB1 1PT. Email: Sarah.Redsell@anglia.ac.uk

Dr Jennie Rose, (JR), Research Fellow, Faculty of Health, Social Care and Education, Anglia Ruskin University. Email: Jennie.Rose@anglia.ac.uk

Dr Stephen Weng, (SW), NIHR Research Fellow, Division of Primary Care, University of Nottingham, Email: Stephen.Weng@nottingham.ac.uk

Dr Joanne Ablewhite, (JA), Research Fellow, Institute of Mental Health, University of Nottingham Innovation Park. Email: joanne.ablewhite1@nottingham.ac.uk

Dr Judy Anne Swift, (JS), Associate Professor of Behavioural Nutrition, Division of Nutritional Sciences, School of Biosciences, University of Nottingham. Email: Judy.Swift@nottingham.ac.uk

Professor A. Niroshan Siriwardena, (ANS), Professor of Primary and Pre-hospital Health Care, Community and Health Research Unit, School of Health and Social Care, University of Lincoln. Email: nsiriwardena@lincoln.ac.uk

Dr Dilip Nathan, (DN), Consultant Paediatrician, Nottingham University Hospitals Trust. Email: Dilip.nathan@nhs.net

Professor Heather Wharrad, (HW), Professor of e-Learning and Health Informatics, School of Health Sciences, University of Nottingham, Email: Heather.Wharrad@nottingham.ac.uk

Mrs Pippa Atkinson, (PA), Lead Health Visitor for Infant Nutrition, Nottingham City Care Partnership. Email: Pippa.Atkinson@nottinghamcitycare.nhs.uk

Mrs Vicki Watson, (VW), Specialist Public Health Dietician, Nottingham City Care Partnership. Email: Vicki.Watson@nottinghamcitycare.nhs.uk

Dr Fiona McMaster, (FM), Senior Lecturer in Public Health, Faculty of Medical Sciences, Anglia Ruskin University Email: Fiona.McMaster@anglia.ac.uk

Dr Rajalakshmi Lakshman, (RL), Consultant in Public Medicine (Lead for Children), Cambridgeshire and Peterborough Public Health Directorate, Shire Hall, Castle Hill, Cambridge, CB3 0AP. Email: Rajalakshmi.Lakshman@mrc-epid.cam.ac.uk

Professor Cris Glazebrook, (CG), Professor of Health Psychology,
Room B12, Institute of Mental Health, University of Nottingham Innovation Park, Triumph Road, Nottingham NG7 2TU. Email: Cris.Glazebrook@nottingham.ac.uk

For peer review only - http://bmjopen.bmj.com/site/about/guidelines.xhtml

ABSTRACT

Objective

To assess the feasibility and acceptability of using digital technology for Proactive Assessment of Obesity Risk during Infancy (ProAsk) with UK Health Visitors (HVs) and parents.

Design

Multi-centre, pre and post intervention feasibility study with process evaluation.

SETTING

Rural and urban deprived settings, UK community care.

Participants

66 parents of infants and 22 HVs.

Intervention

ProAsk was delivered on a tablet device. It comprises a validated risk prediction tool, to quantify overweight risk status, and a therapeutic wheel detailing motivational strategies for preventive parental behaviour. Parents were encouraged to agree goals for behaviour change with HVs who received motivational interviewing training.

Outcome measures

We assessed recruitment, response and attrition rates. Demographic details were collected, and overweight risk status. The proposed primary outcome measure was weight-for-age z-score. The proposed secondary outcomes were parenting self-efficacy, maternal feeding style, infant diet and exposure to physical activity/sedentary behaviour. Qualitative interviews ascertained the acceptability of study processes and intervention fidelity.

Results

HVs screened 324/589 infants for inclusion in the study and 66/226 (29%) eligible infants were recruited. Assessment of overweight risk was completed on 53 infants and 40% of these were identified as above population risk. Weight-for-age z score (SD) between the infants at population risk and those above population risk differed significantly at baseline (-0.67 SD vs 0.32 SD). HVs were able to collect data and calculate overweight risk for the infants. Protocol adherence and intervention fidelity was a challenge. HVs and parents found the information provided in the therapeutic wheel appropriate and acceptable.

Conclusion

Study recruitment and protocol adherence were problematic. ProAsk was acceptable to most parents and HVs but intervention fidelity was low. There was limited evidence to support the feasibility of implementing ProAsk without significant additional resources. A future study could evaluate ProAsk as a HV supported, parent-led intervention.

For peer review only - http://bmjopen.bmj.com/site/about/guidelines.xhtml

Strengths and limitations of this study

This study was the first to examine the feasibility of using the Infant Risk of Overweight Checklist (IROC) prediction algorithm to differentiate between infants at population and above population risk of being overweight.

Qualitative interviews providing both parent and HVs' perspectives on the feasibility of conducting a future RCT and implementing ProAsk within the proposed design were a study strength.

The successful recruitment of some parents from socially deprived areas demonstrated that obesity prevention interventions can be implemented in hard to reach populations.

The main challenges were misinterpretation of participant eligibility by health visitors resulting in less than adequate recruitment and varying levels of intervention fidelity.

Additional information

Trial registration number

NCT02314494

Funding

This work was supported by the Medical Research Council – Public Health Intervention Development Scheme, grant number PHIND 01/14-15.

Authors contribution

The authors have no competing interests.

SR, PI for study and lead author, obtained NHS ethics permission, assembled team, accessed gatekeepers, project managed the study, wrote the first draft of the manuscript.

JR responsible for day to day study coordination at two sites, recruited participants, collected and analysed data, contributed to the drafting of the manuscript.

SW responsible for data processing and analysis, preparing quantitative tables and results, editing, and preparation of manuscript drafts.

JA responsible for day to day study coordination at two sites, recruited participants, collected and analysed data, and contributed to a revision of the manuscript.

JS advised on study design, was involved in data analysis and interpretation of findings and contributed to revision of the manuscript.

ANS advised on study design, was involved in interpretation of findings and contributed to a revision of the manuscript.

DN advised on study design, was involved in interpretation of the findings and contributed to a revision of the manuscript.

For peer review only

HW responsible for the design and development of the ProAsk digital tool, contributed to study design and revision of the manuscript.

PA contributed to the content of the therapeutic wheel and the information that accompanied it. Contributed to the co-ordination of the training for HVs and feedback meetings. Provided local Trust data.

VW contributed to the content of the therapeutic wheel and the information that accompanied it and to revision of the manuscript.

FMc delivered HV training in motivational interviewing, contributed to text within ProAsk digital tool and revision of the manuscript.

RL contributed to study design and revision of the manuscript.

CG contribution to study design, development of ProAsk digital tool, study management and revision of the manuscript.

Data sharing statement

There is unpublished data in relation to the parents' responses to the pre and post questionnaires which can be obtained by contacting the PI. The unpublished qualitative data is being analysed by the authors for a separate publication.

Acknowledgments

We are grateful to the parents and members of the health visiting teams who took part in this study. We particularly wish to thank Catherine Shiels and Fleur Seekins who managed the health visiting service in the study sites.

For peer review only

BACKGROUND

Obesity is a global public health challenge that affects all ages. In 2015, over 42 million children under the age of five were overweight [1]. In the UK in 2015/2016, over a fifth (22.1%) of children aged 4-5 years were either overweight or obese [2], with the highest rates in those living in socioeconomically deprived areas [2, 3] or of Asian or Black ethnicity [2]. Although children are born with genetic predispositions related to weight and growth [4], dietary behaviour is modulated by feeding experience and the family environment [5]. Interventions that address these practices have a role in childhood obesity prevention [6].

Parents and health professionals have called for reliable and valid methods of identifying infants at risk of developing childhood obesity [7-9]. This is possible because the risk factors for childhood overweight and obesity are identifiable antenatally and during infancy [10, 11]. These include parental weight, smoking during pregnancy, birth weight and rapid weight gain; with breastfeeding being moderately protective. Of these, the strongest risk factor for childhood overweight is rapid infant weight gain [12-14]. Between 25%-33% [15, 16] of infants gain weight more rapidly than desirable during the first 6 months of life [17-19] and this risk factor is potentially modifiable if identified during early life. The Infant Risk of Overweight Checklist (IROC) [12, 20], developed using data from the Millennium Cohort Study (MCS), [21] and externally validated using the Avon Longitudinal Study of Parents and Children (ALSPAC) data [22], operationalises an algorithm to predict an infant's future risk of being overweight. IROC [12, 20] offers the opportunity to identify infants at greatest risk of overweight in clinical practice.

Interactive digital technology can support complex and sensitive discussions between health professionals and patients [23-25]. It is also suited to delivering personalised information about health status and risk [26]. Potential behaviour change can be raised through the neutrality of a technological device without increasing anxiety [27]. Proactive Assessment of Obesity Risk during Infancy (ProAsk) is a novel, interactive digital intervention designed to equip Health Visitors (HVs) with an individual infant's risk of future overweight, whilst also supporting discussions with parents. It incorporates the IROC [12, 20] and a therapeutic wheel which has previously been found to be an acceptable format for cancer patients [28]. The therapeutic wheel comprises an interactive graphic detailing strategies for preventive behaviour using supportive and strengths-based prompts. The content of the therapeutic wheel is based on the behavioural strategies identified in a systematic review of interventions that reduce the risk of childhood obesity in early life [6]. ProAsk is designed to be delivered alongside Motivational Interviewing (MI) which has been successfully used to improve health behaviours including diet and physical activity for parent-child dyads [29, 30].

This is the first intervention designed to identify an infant's risk of overweight and to provide parents of infants at greatest risk with strategies for prevention. There is no evidence for the effectiveness of this approach to draw on to guide our study design, and so, in line with the MRC's framework for the of development and evaluation of complex interventions, [31] a feasibility study was required.

METHOD

STUDY AIM AND OBJECTIVES

This study aimed to examine the feasibility and acceptability of undertaking a randomised controlled trial (RCT) of ProAsk with UK HVs and parents. We used the ADePT framework [32] to examine the methodological feasibility, and to address the study's objectives which were:

For peer review only - http://bmjopen.bmj.com/site/about/guidelines.xhtml

(i) To assess recruitment, response and attrition rates for parents/legal guardians/carers of infants;

(ii) To determine the proportion of infants calculated as at risk using ProAsk at baseline to inform a sample size calculation for a future RCT;

(iii) To evaluate the feasibility of HV delivery of the ProAsk intervention to eligible parents/legal guardians/carers, including an assessment of intervention fidelity and protocol adherence;

(iv) To determine the acceptability and feasibility of the proposed primary and secondary outcomes measures.

Design

Multi-centre, pre and post intervention feasibility study with process evaluation.

Participants and recruitment

ProAsk was delivered by HVs to parents in four study sites in two localities, in urban and rural deprived areas within the UK. One day's training in recruitment processes, ProAsk and MI [33] was delivered to members of the HV teams (N=47). Delivering the intervention required the skills of a HV but to ensure the team understood study processes we trained nursery nurses (N=12), an administrator (N=1), student nurses (N=3), managers (N=3), together with HVs (N=28). The MI training was delivered by a member of the motivational interviewing network of trainers (MINT) and comprised interactive and experiential activities [34] including agenda-mapping and reflective listening. HVs were also offered half a day top-up training in MI during the study period and six HVs from two sites attended. Recruitment commenced on 22nd April 2015 in two sites and 12th June in the other sites, and ended on 30th November 2015.

The local NHS Trusts HV managers estimated that the numbers of births within the study areas over a three month period was 700. The National Institute for Health Research- Clinical Research Network (NIHR-CRN) estimated that HVs could recruit up to 30% of eligible parents to the study providing a pragmatic sample of approximately N=100 infants over a three month period.

Parents of infants aged 6-8 weeks were eligible to take part. Exclusion criteria were (a) infant with known medical conditions requiring special diets, (b) mother with diagnosis of post-natal depression (PND) or score of moderate PND or above on health visitor applied screening tools (EPDS >13) [35] (PHQ-9 >10) [36] or anxiety (GAD-7 score >10) [37], (c) infant born before 32 weeks gestation (d) infant birth weight below 2nd centile (e) parent with insufficient English to complete the questionnaires in the absence of face-to-face translation.

Health visitors were asked to identify all infants attending for their 6-8 week check over a three month period or until the sample size was met. They identified eligible parents at a routine infant check and approached them with information about the study. HVs recorded reasons for exclusion, response to approach, and reasons for refusal on a log sheet. Interested participants gave their permission to be contacted by the researchers by telephone to arrange a home visit, where informed written consent was obtained. Where parents did not respond to the initial phone call the researchers made two further calls at different times of the day. At the end of the study a purposively diverse sample of parent participants were invited to take part in qualitative interviews together with all of the HVs.

For peer review only - http://bmjopen.bmj.com/site/about/guidelines.xhtml

Figure 1 shows the per protocol study regimen.

INSERT FIGURE 1

Ethics and research governance permissions

Permission to conduct the study was provided by East of England (Essex) NHS Research Ethics Committee on 26th February 2015 (Reference number 15/EE/0011). Research governance permissions were provided by the two NHS Trusts covering the study localities.

Intervention

HVs used a hand-held device (tablet) to deliver ProAsk to parents when their infants were three months old. This involved entering the IROC [12] items (baby birth weight and length, current weight, maternal and paternal height and weight, maternal smoking status during pregnancy, and breastfeeding) into ProAsk which then calculated the infant's risk status using the World Health Organisation (WHO) growth charts [38]. This was displayed on the tablet screen as either "Your baby's risk of being above a healthy weight is the same as other babies" (population risk) or "Your baby's risk of being above a healthy weight is more than other babies" (above population risk). Responses were stored on the password protected tablet. Two tablets were provided per site. Problems with internet access at two sites resulted in an amendment to the data extraction method and HVs were asked to screen shot the IROC result for transfer to the research team.

HVs were asked to offer parents who received the above population risk message an opportunity to explore the therapeutic wheel (Figure 2). This interactive graphic promoted evidence-based behaviour change strategies [39] in four areas: active play; milk and solid foods; sleeping and soothing; and infant feeding cues. It prompted HVs to use a motivational approach [33] to build parental self-efficacy for agreed behaviour goals, which were recorded on leaflets left in the home as cues to action for behavioural change.

INSERT FIGURE 2

Measures and data collection

We recorded the number of participants identified by the NHS Child Health records and compared this to the numbers identified by the HVs. We also recorded the number of participants who were eligible, approached and recruited as well as the return of the follow up measures. A summary of the feasibility data collection measures can be found in Supplementary file 1. We collected data on the acceptability and feasibility of collecting data on the proposed primary and secondary outcome measures. The proposed primary outcome measure was weight-for-age z-score, using the WHO growth charts. The proposed secondary outcomes were parenting self-efficacy, maternal feeding style, infant diet and exposure to physical activity/sedentary behaviour.

Demographic details, ethnicity and information about family size were collected at baseline (infant age two months) via a self-report questionnaire completed by parents. Details of the infants IROC score were recorded at three months. Infant anthropometric data, details of infant feeding (breast or formula milk or both) and validated measures of parenting self-efficacy [40, 41] and maternal feeding style (Infant Feeding Questionnaire (IFQ)) [42] were collected by self-report questionnaire at infant age 2 months (baseline) and 6 months (follow-up). In addition, exposure to opportunities for

For peer review only - http://bmjopen.bmj.com/site/about/guidelines.xhtml

physical activity and sedentary behaviour was recorded by parents at baseline and follow-up as time spent unrestricted on tummy, and restricted in a baby seat, car seat or pushchair.

Parents were interviewed about the acceptability of ProAsk, study processes, including recruitment, and intervention fidelity. HVs were interviewed to explore their experiences of recruiting parents to the study and conducting the ProAsk Assessment. They were also asked about environmental factors such as the compatibility of ProAsk with existing workplace goals, organisational barriers and support for the intervention, and their views on the quality of training provided by the team. Interviews lasting up to 90 minutes, were conducted face-to-face and over the telephone, and recorded using a digital Dictaphone.

DATA ANALYSIS

Recruitment, response and attrition rates, demographic details, weight-for-age z score, and overweight risk status (population risk vs. above population risk at 10% risk threshold) were analysed using descriptive statistics via STATA 13 MP4.

Audio data from qualitative interviews with parents and HVs were transcribed verbatim and transcripts were imported into Nvivo software for sorting, coding and categorising. Data relating to relevant methodological issues were subject to thematic content analysis using the method outlined by Boyatzis (1998) [43]. Verbatim quotes illustrate the themes.

RESULTS

The results for each of the ADePT framework's [32] methodological issues are summarised in Table 1, together with strategies for improving a future study design. The demographic data are detailed in Table 2.

INSERT TABLES 1 AND 2

The results of the thematic content analysis of the parent (N=12) and HV (N=15) interviews are presented in Tables 3a and 3b.

INSERT TABLES 3A AND 3B

SAMPLE SIZE CALCULATIONS

A total of 324 infants were screened by the HVs during a routine a 6-8 week check and consent was obtained from 66 parent-infant dyads (20%). An overweight risk assessment was completed for 56/66 infants and the data transferred to the research team for 53 of these. This showed that 40% infants were above population risk. Sufficient data were collected to inform a sample size calculation but our findings suggest that more attention to study design is needed prior to future evaluation of ProAsk.

ELIGIBILITY

The study flow chart presented in Figure 3 details participant eligibility and the reasons for exclusion. The number of 6-8 week checks logged by the NHS Child Health Records during the extended recruitment phase was 589 which was fewer than the 700 estimated in three months by the NHS Trusts. HVs screened only 324 of these (45%) during the extended recruitment period (3 to 5.5 months for one locality and seven for the other).

For peer review only - http://bmjopen.bmj.com/site/about/guidelines.xhtml

INSERT FIGURE 3

In the HV interviews, language was identified as a major barrier to participant eligibility (N=9), particularly in one site (Table 2a, N=7). HVs (N=8) were also concerned about referring parents with mental health, safeguarding or domestic violence issues.

> ***HV N20:*** *It was the language barrier really. I'd say one hundred percent or ninety nine percent of my parents are non-English speaking so obviously without an interpreter.*

> ***HV C37:*** *Because there were other issues around perhaps, safeguarding, in need, other agencies working with that family, and yet something else for them to have to deal with.*

Recruitment

The recruitment target of N-100 infants in three months was not met. The most common reasons for parents declining were: parents not interested (N=28) and parents lacked time (N= 21). The sample contained more than the expected number of mothers with degree level education. The Income Deprivation Affecting Children Index (IDACI) [44], which measures area deprivation based on post-code, for participants with completed risk assessment showed that more (33%) of the participants recruited were from the two lower quintiles than from the two upper quintiles (25%) (Table 2).

In total, 22/28 HVs who received training took part in the study (the remaining HVs were transferred, elsewhere, or on sickness or maternity leave). Most HVs interviewed took part at the request of their managers. Workload was identified as a barrier to parent recruitment by 5/15 HVs interviewed. Six reported being wary of raising the study with parents (Table 3a).

Of the 12 parents interviewed, 11 found the study recruitment processes acceptable and 10 felt well-informed. Seven parents participated because of concerns about their own weight and a further seven did so for altruistic reasons. Eleven parents were willing to be randomised for a future trial around identification and intervention with infants at future risk of overweight.

Consent

Of the 138 parents who gave permission to be contacted by the researchers, 66 (48%) provided written consent (Figure 3). Interviews with HVs suggested that one reason for the low conversion rate to written consent was that parents were wary of accepting a telephone call or arranging a home visit with unknown researchers.

> ***HV N9:*** *So I think that then when I said someone else would come in after me, some families were not keen to take part. Half our battle is for us to get in, then when I said someone else, I found that was hard.*

Adherence to intervention

In total, 56/66 infants had their overweight risk score calculated. Interviews with the HVs suggested that the main reason that ten parents did not receive their risk assessment was that they were not at home when the HV came to deliver ProAsk.

> ***HV C22:*** *A couple of them I've been to their house at the designated time and they haven't been there so I haven't revisited them. Because you know, if you go out and see them and*

For peer review only - http://bmjopen.bmj.com/site/about/guidelines.xhtml

> *they're not there what do you do? You perhaps have to chase them up but to honest I haven't had the time.*

An important element was for HVs to feedback the overweight risk score to parents, but four parents were not aware of having received this feedback or were uncertain as to what it meant for them. Four HVs interviewed reported difficulties feeding back to parents the overweight risk score.

Although goal-setting and follow-up contact was recommended for infants identified as being above population risk of overweight, this did not always take place. Goal-setting around behaviour change was recorded for only five of the 21 parents whose infants were at above population risk. HV interviews confirmed that of the 11 HVs who had conducted a ProAsk assessment and were interviewed, seven had shown parents all elements of the wheel rather than focussing on one specific area. There was little evidence that MI had been used to facilitate goal setting and behaviour change. Three of the HVs interviewed had used the therapeutic wheel to provide information to all participants, irrespective of their risk score status.

> ***HV C22:*** *I know when I did the actual wheel, if you like, you said to discuss one topic, we ended up discussing them all. Because all of those topics are covered in health visiting anyway, to me it didn't feel right that we talked about diet without exercise and feeding cues.*

Intervention acceptability

A total of 88 parents declined participation. Eight out of 12 parent participants found ProAsk acceptable and were positive about its digital functionality. One parent expressed disappointment with ProAsk. There was evidence that ProAsk helped to engage parents and avoided information overload.

> ***Parent C8:*** *I thought the information in there was really nice and visual actually, because sometimes you can hear a lot of information and it's sort of difficult to absorb it and there was quite a lot of it, it was nice to have something in front of you as well as you were having that discussion. A sort of a visual prompt you could refer back to.*

Six out of 28 HVs did not take part in the study. Eight HVs expressed initial concerns about the unintended consequences of communicating overweight risk status, particularly at infant age 3 months which some considered too early for personalised risk communication.

> ***HV N5****: I mean when I first heard about the research I was quite concerned initially because I had visions of mothers sort of starving their babies that's more of a risk than over feeding a baby at that sort of very early age, delicate age when they're so young.*

However, HVs also recognised the potential benefits of early intervention to prevent overweight with seven stating they found the therapeutic wheel engaging to use and containing useful information. One HV suggested that the intervention was disappointing.

Outcome assessment

Table 2 shows the demographic and participant characteristics at baseline stratified by overweight risk status. At the 10% risk threshold, 32 (60%) infants were at average population risk and 21 (40%) were above. There was a statistically significant difference in birth weight (3.28 vs 3.86) and weight-for-age z score (-0.67 vs 0.32) between the infants that were at population risk and those above

For peer review only - http://bmjopen.bmj.com/site/about/guidelines.xhtml

population risk at baseline. There was also a significant difference in paternal BMI (26.5 vs 30.1kg/m^2) but not pre-pregnancy maternal BMI or smoking status.

Selection of outcomes

The parent self-report measures were completed fully by 85% of respondents. However, there was missing data for infant length and head circumference because these measures are not routinely recorded in parent-held infant records. Cronbach's alpha for the parenting self-efficacy [40, 41] and maternal feeding style (Infant Feeding Questionnaire (IFQ)) [42] measures all exceeded >0.5, indicating acceptable internal consistency [45, 46].

Four parents described how completing the baseline questionnaire had prompted them to change their behaviour around infant opportunities for active play and sedentariness. This was an unexpected measurement effect.

> ***Parent C16:*** *When I filled in the questionnaire, at least the first time I filled them in, there were a few that made me think about how I could change it. For example there was a question about how much tummy time the baby gets. And I'd never really thought of that as a form of exercise, which I then started to do. And it made me more aware of trying to get my son that tummy time. It made me think it's not all about what they eat, it's about, well exercising the calories off.*

Study retention

In total, 34/66 parents returned the follow-up questionnaire at six months (51% retention rate). 15 (71%) of parents whose infants were at higher risk returned their follow up questionnaire. Three parents had not received the intervention. All parents (N=12) invited to participate in post-study interviews agreed to take part.

Logistics of multicentre trial

One site recruited more parents than the others (see Table 1). HVs from this site were able to overcome the challenges that occurred in the early stages of the project through team working and reaching out to the researcher for support. The HVs from the other sites talked about the teams or their own resistance to the study because they felt their geographical area was unsuitable for the study.

> ***HV C43:*** *Our administrator was brilliant; I knew you were on the end of the phone; I had support from my peers, if we didn't know how to do something we worked it out between us.*

> ***HV N20.*** *It was just said that the area has been chosen so that was fine. I just think that it was the wrong area, absolutely totally the wrong area.*

Components of protocol working together

The low level of fidelity suggests there were incompatibilities between the risk assessment and preventative strategies within ProAsk. HVs found it difficult to find the time for additional home visits to complete the behaviour change aspect of the intervention.

For peer review only - http://bmjopen.bmj.com/site/about/guidelines.xhtml

***HV: C22** I have to say we are a bit stretched for time, we're short of staff, and it is another visit that we have to fit in on top of everything else. So from that point of view, it was a bit stressful I suppose.*

DISCUSSION

The aim of the study was to determine the feasibility and acceptability of conducting an RCT of ProAsk. Parents and HVs found the study processes acceptable and ProAsk engaging. However, HVs had reservations about assessing and communicating overweight risk to parents of young infants. Overall recruitment to the study was lower than expected. Poor conversion of potential participants to consent resulted in the study failing to meet the recruitment target. There were problems with protocol adherence and intervention fidelity, with some parents not receiving all elements of the intervention.

This study was conducted in areas identified as being socially deprived because childhood obesity is more prevalent [2, 3]. Whilst recruitment was disappointing, the IDACI [44] scores for the infant participants show that more socioeconomically deprived households were included. The fact that 40% of infants recruited were assessed as being at risk of overweight and that 71% of these returned their follow-up questionnaire demonstrates that our target group was sampled. Recruiting participants from these areas is known to be challenging [47]. Other research studies have used opt-in [48] or financial incentives [49] to improve recruitment in socially disadvantaged areas. However, the potential for parental stigmatisation [50] make such strategies less applicable to studies of overweight prevention. Language was a significant barrier to recruitment, and future research will need to ensure that interpreting and translation service are resourced

The sample contained a relatively high proportion of participants with degree level education. HV logs of 6-8 week visits and interviews with HVs indicated that some groups of parents such as those with a history of mental health concerns, were not approached about the study, even if they were eligible for participation, because of concerns about their ability to deal with study burden. There is evidence from other settings that professional gatekeepers do not approach all participants eligible for healthcare research [51-54]. To inform our future study design we need to understand how to improve participant identification and recruitment. Therefore, we are currently conducting a study to identify UK HVs' perspectives on the enablers and barriers to research recruitment. It will report in late 2017.

HVs were wary about risk assessment and some had anxieties about raising the topic of weight with parents. Ten infants did not receive the risk assessment which was explained in terms of parents being unavailable but may also reflect HVs hesitancy. Problems with the technology in the field resulted in the data from three infants not transferring from tablet devices to the research team. An alternative or complementary approach could be to use routine clinical data from both parents and children for anthropometrics. However, this could potentially reduce parental engagement and understanding of the activity of overweight risk assessment. The next phase of the study will explore whether HVs are best placed to undertake risk assessment discussions with parents and if so what training will ensure parents receive accurate information. Most HVs showed parents all the preventative information available on the therapeutic wheel rather than guiding them towards their own goals for behaviour change. HV service schedule advises them to use a motivational approach [55] but it seemed to be challenging for HVs to use this approach in this study. Additional bespoke

For peer review only - http://bmjopen.bmj.com/site/about/guidelines.xhtml

training building on their existing knowledge of MI, would help HVs to deliver the behaviour change components of the intervention as intended.

Parents found ProAsk engaging and the use of digital technology acceptable. Other studies have shown similar acceptability and engagement with digital interventions resulting in improved retention of information and advice that had not been retained following a consultation [56, 57]. The ProAsk therapeutic wheel could be adapted to provide accessible digital information for parents and carers which would address their request for on-going information in digital format.

The researchers were able to collect data on outcomes of interest from parents at the times specified in the protocol. A minority of parents reported that the questionnaire items around infant activity and sedentary behaviour led them to consider behaviour change which may be a contamination risk for a future RCT. Respondent agreement between the validated outcome measures varied from poor to good, with higher levels of internal consistency in the follow up questionnaire.

CONCLUSIONS

The study identified significant problems with study recruitment and protocol adherence. Many of these problems could be addressed by employing dedicated researchers to screen and recruit participants. Although the intervention was acceptable to most parents and HVs interviewed, the fidelity of delivery was disappointing. There was limited evidence to support the feasibility of adding ProAsk to HV's role without significant additional resources. A future study could evaluate ProAsk as a stand-alone, parent led-digital intervention or as a HV supported, parent-led intervention.

REFERENCES

1. **UNICEF, WHO, The World Bank: Joint Child Malnutrition Estimates - Levels and Trends: Key findings of the 2016 edition**
2. NHS Digital: **National Child Measurement Programme**. In.; 2015/2016.
3. Shrewsbury V, Wardle, J: **Socioeconomic status and adiposity in childhood: a systematic review of cross-sectional studies 1990-2005**. *Obesity* 2008, **16**(2):275-284.
4. Elks CE, Loos RJ, Hardy R, Wills AK, Wong A, Wareham NJ, Kuh D, Ong KK: **Adult obesity susceptibility variants are associated with greater childhood weight gain and a faster tempo of growth: the 1946 British Birth Cohort Study**. *Am J Clin Nutr* 2012, **95**(5):1150-1156.
5. Dubois L, Ohm, KK, Girard, M, Tatone-Tokuda, F, Pérusse, D, Hjelmborg, J, Skytthe, A, Rasmussen, F, Wright, MJ, Lichtenstein, P, Martin, NG: **Genetic and Environmental Contributions to Weight, Height, and BMI from Birth to 19 Years of Age: An International Study of Over 12,000 Twin Pairs**. *PLoS ONE* 2012, **7**(2):e30153.
6. Redsell S, Edmonds B, Nathan D, Siriwardena AN, Swift J, Weng SF, Glazebrook C: **Systematic review of behavioural and non-behavioural interventions, delivered during infancy and the antenatal period, that reduce the risk of overweight and obesity in childhood** *Matern Child Nutr* 2015, **April** (DOI: 10.1111/mcn.12184).
7. Redsell S, Atkinson PJ, Nathan D, Siriwardena AN, Swift JA, Glazebrook C: **Parents' beliefs about appropriate infant size, growth and feeding behaviour: implications for the prevention of childhood obesity**. *BMC Public Health* 2010, **10**(1):711.
8. Redsell S, Atkinson PJ, Nathan D, Siriwardena AN, Swift JA, Glazebrook C: **Preventing childhood obesity during infancy in UK primary care: a mixed-methods study of HCPs' knowledge, beliefs and practice** *BMC Family Practice* 2011, **12**:54.
9. Redsell S, Swift JA, Nathan D, Siriwardena AN, Atkinson P, Glazebrook C: **UK health visitors' role in identifying and intervening with infants at risk of developing obesity**. *Matern Child Nutr* 2013, **9**(3):396-408.

For peer review only - http://bmjopen.bmj.com/site/about/guidelines.xhtml

10. Weng SF, Redsell SA, Swift JA, Yang M, Glazebrook CP: **Systematic review and meta-analyses of risk factors for childhood overweight identifiable during infancy**. *Archives of Disease in Childhood* 2012, **97**(12):1019-1026.
11. Woo Baidal JA, Locks LM, Cheng ER, Blake-Lamb TL, Perkins ME, Taveras EM: **Risk Factors for Childhood Obesity in the First 1,000 Days: A Systematic Review: A Systematic Review**. *American Journal of Preventive Medicine* 2016, **50**(6):761-779.
12. Weng S, Redsell, SA, Nathan, D, Swift, JA, Yang, M, Glazebrook, C: **Developing an algorithm to estimate overweight risk in childhood from predictors during infancy**. *Pediatrics,* 2013, **132**(2):e414-e421.
13. Elks CE LR, Sharp SJ, Langenberg C, Ring SM, Timpson NJ, Ness AR, Davey Smith G, Dunger DB, Wareham NJ, Ong KK.: **Genetic markers of adult obesity risk are associated with greater early infancy weight gain and growth**. *PLoS Med* 2010, **7**(5):e1000284.
14. Druet C, Stettler N, Sharp S, Simmons RK, Cooper C, Smith GD, Ekelund U, Levy-Marchal C, Jarvelin MR, Kuh D, Ong KK: **Prediction of childhood obesity by infancy weight gain: an individual-level meta-analysis**. *Paediatric and Perinatal Epidemiology* 2012, **26**(1):19-26.
15. Ekelund U, Ong K, Linne Y, Neovius M, Brage S, Dunger DB, Wareham NJ, Rossner S: **Upward weight percentile crossing in infancy and early childhood independently predicts fat mass in young adults: the Stockholm Weight Development Study (SWEDES)**. *American Journal of Clinical Nutrition* 2006, **83**(2):324-330.
16. Hui L, Schooling CM, Leung SS, Mak KH, Ho LM, Lam TH, Leung GM: **Birth weight, infant growth, and childhood body mass index: Hong Kong's children of 1997 birth cohort**. *Archives of Pediatrics & Adolescent Medicine* 2008, **162**(3):212-218.
17. Ong K, Loos R: **Rapid infancy weight gain and subsequent obesity: systematic reviews and hopeful suggestions**. *Acta Paediatr* 2006, **95**:904 - 908.
18. Baird J, Fisher D, Lucas P, Kleijnen J, Roberts H, Law C: **Being big or growing fast: systematic review of size and growth in infancy and later obesity**. *BMJ* 2005, **331**(7522):929.
19. Monteiro P, Victora CG: **Rapid growth in infancy and childhood and obesity in later life--a systematic review**. *Obes Rev* 2005, **6**(2):143-154.
20. Redsell SA WS, Swift JA, Nathan D, Glazebrook C. : **Validation, optimal threshold determination and clinical utility of the Infant Risk of Overweight Checklist (IROC) for early prevention of child obesity** *Childhood Obesity* 2016, **12**(3):202-209.
21. **Centre for Longitudinal Studies: Millenium Cohort Study**
22. **Avon Longitudinal Study of Parents and Children (ALSPAC)** [http://www.bristol.ac.uk/alspac/]
23. Ferguson M, Brandreth M, Brassington W, Leighton P, Wharrad H: **A Randomized Controlled Trial to Evaluate the Benefits of a Multimedia Educational Program for First-Time Hearing Aid Users**. *Ear & Hearing* 2016, **0**:1-14.
24. Raaff C, Glazebrook C, Wharrad H: **A systematic review of interactive multimedia interventions to promote children's communication with health professionals: implications for communicating with overweight children**. *BMC Medical Informatics and Decision Making* 2014, **14**(1):8-8.
25. Manning JC, Latif A, Carter T, Cooper J, Horsley A, Armstrong M, Wharrad H: **'Our Care through Our Eyes': a mixed-methods, evaluative study of a service-user, co-produced education programme to improve inpatient care of children and young people admitted following self-harm**. *BMJ open* 2015, **5**:e009680-e009680.
26. Widmer RJ, Allison, T. G., Keane, B., Dallas, A., Lerman, L. O., Lerman, A.: **Using an online, personalized program reduces cardiovascular risk factor profiles in a motivated, adherent population of participants**. *American heart journal* 2014, **167**(1):93-100.
27. Glazebrook C, Garrud P, Avery A, Coupland C, Williams H: **Impact of a multimedia intervention "Skinsafe" on patients' knowledge and protective behaviors**. *Preventive Medicine* 2006.
28. Gordon D: **Breaking Barriers: Management of Cancer-related Pain (Educational CD ROM)** *The Clinical Journal of Pain* 2009, **25**(4):351.

For peer review only - http://bmjopen.bmj.com/site/about/guidelines.xhtml

29. Borrelli B, Tooley, EM, Schott-Sheldon, LAJ: **Motivational interviewing for parent-child health interventions: a systematic review and meta-analysis**. *Pediatric dentistry* 2016, **37**(3):254-265.
30. Resnicow K, McMaster, F, Bocian, A, Harris, D, Zhou, Y, Snetselaar, L, Schwartz, R, Myers, E, Gotlieb, J, Foster, J, Hollinger, D, Smith, K, Woolford, S, Mueller, D, Wasserman. RC: **Motivational Interviewing and Dietary Counseling for Obesity in Primary Care: An RCT.** *Pediatrics* 2015, **135**(4):649-657.
31. Craig P, Dieppe P, Macintyre S, Michie S, Nazareth I, Petticrew M: **Developing and evaluating complex interventions: the new Medical Research Council guidance**. *BMJ* 2008, **337**.
32. Bugge C, Williams B, Hagen S, Logan J, Glazener C, Pringle S, Sinclair L: **A process for Decision-making after Pilot and feasibility Trials (ADePT): development following a feasibility study of a complex intervention for pelvic organ prolapse.** *Trials* 2013, **14**:353.
33. Miller WR, Rollnick S: **Motivational Interviewing: Helping People Change**, 3 edn; 2013.
34. Edwards E, Stapleton, P, Williams, K, Ball, L: **Building skills, knowledge and confidence in eating and exercise behaviour change: Brief motivational interviewing training for healthcare providers**. *Patient Education and Counseling* 2015, **98**(5):674-676.
35. Cox J, Holden, JM, Sagovsky, R. : **Detection of postnatal depression: Development of the 10-item Edinburgh Postnatal Depression Scale**. *British Journal of Psychiatry* 1987, **150**(782-786).
36. Spitzer RL KK, Williams JB, et al. : **Validation and utility of a self-report version of PRIME-MD: the PHQ primary care study**. *JAMA* 1999, **282**(19):1737-1744.
37. Spitzer RK, K, Williams, JBW, Löwe, B,: **A brief measure for assessing generalized anxiety disorder: The GAD-7**. *Archives of Internal Medicine* 2006, **166**(10):1092-1097.
38. World Health Organisation WHO: **Child Growth Standards - Weight Velocity**. In.
39. Redsell SA, Edmonds B, Swift JA, Siriwardena AN, Weng S, Nathan D, Glazebrook C: **Systematic review of randomised controlled trials of interventions that aim to reduce the risk, either directly or indirectly, of overweight and obesity in infancy and early childhood**. *Maternal & Child Nutrition* 2016, **12**(1):24-38.
40. Johnston C, Mash, E.J.: **A measure of parenting satisfaction and efficacy**. *Journal of Clinical Child Psychology* 1989, **18**:167-175.
41. Gilmore L, Cuskelly, M.: **Factor structure of the Parenting Sense of Competence Scale using a normative sample**. *Child Care, Health & Development* 2009, **38**:48-55.
42. Baughcum A, Powers, SW, Johnson, SB, Chamberlin, LA, Deeks, CM, Jain, A, Whitaker, RC: **Maternal feeding practices and beliefs and their relationships to overweight in early childhood**. *J Dev Behav Pediatr* 2001, **22**(6):391-408.
43. Boyatzis R (ed.): **Transforming Qualitative Information: Thematic Analysis and Code Development**. Thousand Oaks, California: Sage Publications; 1998.
44. HM Government Department for Communities and Local Government: **Income Deprivation Affecting Children Index (IDACI)**. In.; 2015.
45. Bowling A: **Research Methods in Health: Investigating health and health services**. Buckingham: Open University Press; 2002.
46. Schmitt M: **Uses and abuse of coefficient alpha.** *Psychol Assessment* 1996, **8**:350-353.
47. Emmel N, Hughes K, Greenhalgh J, Sales A: **Accessing socially excluded people - Trust and the gatekeeper in the researcher-participant relationship**. *Sociological Research Online* 2007, **12**(2).
48. Parry O, Bancroft, A, Gnich, W, Amos, A.: **Nobody home? Issues of respondent recruitment in areas of deprivation** *Critical Public Health,* 2011, **11**(4):305-317.
49. Jennings CG, MacDonald TM, Wei L, Brown MJ, McConnachie L, Mackenzie IS: **Does offering an incentive payment improve recruitment to clinical trials and increase the proportion of socially deprived and elderly participants?** *Trials* 2015, **16**:80.
50. Puhl RM, Heuer, C. A.: **Obesity stigma: important considerations for public health**. *American Journal of Public Health* 2010, **100**(6):1019-1028.
51. Mytton J IJ, Manns S, Stevens T, Mulvaney C, Blair P, Powell J, Potter B, Towner E, Emond A, Deave T, Thomas J, Kendrick D, Stewart-Brown S: **The feasibility of using a parenting**

For peer review only

programme for the prevention of unintentional home injuries in the under-fives: a cluster randomised controlled trial. *Health Technology Assessment* 2014, **18**(3).

52. Preston NJ FM, Walshe CE, et al.: **Strategies Designed to Help Healthcare Professionals to Recruit Participants to Research Studies**: John Wiley & Sons, Ltd; 2016.
53. Bonevski B, Randell M, Paul C, Chapman K, Twyman L, Bryant J, Brozek I, Hughes C: **Reaching the hard-to-reach: a systematic review of strategies for improving health and medical research with socially disadvantaged groups**. *BMC Med Res Methodol* 2014, **14**:42.
54. Hughes-Morley A, Young B, Waheed W, Small N, Bower P: **Factors affecting recruitment into depression trials: Systematic review, meta-synthesis and conceptual framework**. *Journal of affective disorders* 2015, **172**:274-290.
55. NHS Constitution: **National Health Visiting Service Specification**. In.; 2014/2015.
56. Ferguson M, Brandreth, M., Brassington, W. Wharrad, H.: **Information retention and overload in first-time hearing aid users: an interactive multimedia educational solution.** . *American Journal of Audiology* 2015, **24**:329-322.
57. Szeszak S MR, Love A, Langmack G, Wharrad H, Dineen RA: **Animated educational video to prepare children for MRI without sedation: evaluation of the appeal and value.** . *Pediatric Radiology* 2016, **46**(12):1744-1750.

For peer review only

For peer review only - http://bmjopen.bmj.com/site/about/guidelines.xhtml

TABLE 1 SUMMARY OF METHODOLOGICAL ISSUES

Methodological issue	Findings	Evidence	Strategies for improvement
1. Did the feasibility study allow a sample size calculation for the main trial?	Response rate less than expected but absolute numbers potentially allow for sample size calculation for main trial once a suitable design has been determined.	See Figure 3. 66/324 (20%) eligible infants were recruited.<br>56/66 infants had an IROC assessment, but data from three assessments was missing due to data transfer errors, resulting in 53 complete assessments.<br>40% of infants assessed for overweight risk were above population risk.	Allow for longer recruitment time.<br>Allow for more recruitment sites.
2. What factors influenced eligibility and what proportion of those approached were eligible?	Fewer infants attending for 6-8 week check in the study areas.<br>High number of screened infants were reported not to have met the inclusion/exclusion criteria (see Figure 3).	HV Managers at the NHS Trusts estimated there would be 700 infants born into the study areas within a three month period. NHS Child Health Records showed 589 estimated potential participants in the study period of which 324 (45%) were identified by HVs. It is not known how many of the infants who were unrecorded by the HVs might have been eligible for the study. HVs recorded screening 324 (55%) parents. Of these, 226 (70%) were recorded as eligible for the study.	In the UK it is not possible to employ dedicated researchers in one organisation to directly recruit participants to studies being undertaken in another organisation. Therefore participant identification has to be undertaken via a gatekeeper employed by the organisation where the research is taking place. However, once participants are identified dedicated researchers can recruit directly (as was undertaken in this study). Investment in additional training in participant identification is required for HVs. This would require investment from the HVs employing Trusts in terms of time for training activities and continuing professional development.<br>Researcher to screen all participants for eligibility.<br>Direct patient recruitment but this may

For peer review only - http://bmjopen.bmj.com/site/about/guidelines.xhtml

For peer review only

			not enable engagement of the most deprived families with whom HVs have regular contact.
3. Was recruitment successful?	Recruitment target not met despite extending the recruitment period extended from 3 to 5 months.<br>Challenges at individual and team level.	66 parents recruited in 5.5 months and 7 months.<br><br>Not all HVs who were trained took part in the study (22/28). Some did not recruit parents to the study.	Funding for translation and interpreting services.<br>Pre-project discussions about the impact of professional gatekeeping with HVs.<br>Further research is needed to identify barriers and enablers to research recruitment by HVs so that evidence-based interventions to improve recruitment can be developed.
4. Did eligible participants consent?	Low conversion to consent.	226 infants were eligible<br>88 (38%) parents refused to participate.<br>138 parents were identified as being interested in the study. A failure of communication between researchers and some HVs meant that 16 parents who had given their permission to be contacted were not followed up. Researchers were unable to get responses to their telephone calls from 22% (30/138) of parents who initially expressed interest in the study.<br>66 (48%) consented to participate.	Improve communication pathways between HVs and research team.<br>Research team more visible in child health clinics to ensure they are recognisable to parents.
5. Were participants	Not assessed		

For peer review only - http://bmjopen.bmj.com/site/about/guidelines.xhtml

successfully randomised and did randomisation yield equality in groups?			
6. Were blinding procedures adequate?	Not assessed		
7. Did participants adhere to the intervention?	Not all parents had a ProAsk assessment/viewed the therapeutic wheel.	56/66 infants received the risk assessment. Interviews with HVs revealed that all 10 parents who did not receive an assessment were not at home when they called. 21/53 were found to be at above population risk but only five of these received the therapeutic wheel intervention per protocol. Process evaluation revealed that N=4 parents received the assessment but did not receive the risk score feedback.	Better engagement with HVs around the project aims and objectives, and provision of HV time for ongoing engagement with and support from research team about intervention delivery.<br><br>Audit of study processes by research staff and feedback to HVs to increase adherence to the intervention. Increase accessibility to research team for participants with concerns/questions.
8. Was the intervention acceptable to the participants?	Some parents and HVs didn't take part in the study.<br><br>Low number of parents consenting suggest some were concerned about the intervention	6/28 HVs declined to take part in the study. Qualitative interviews suggests they were wary of raising issue of infant weight but adjusted their practice to incorporate ProAsk.<br>88 (38%) parents refused to take part following initial interest. Parent participants found the intervention acceptable.	Determine in further qualitative analysis the particular issues or components of the intervention that HVs were uncomfortable with.
9. Was it possible to calculate intervention costs and duration?	Not assessed		
10. Were outcome assessments completed	HVs were able to complete risk score on 56 participants.<br><br>Follow-up questionnaire completion rate low.	See Table 2 for more details of outcome data.<br><br>34/66 parents completed follow-up questionnaire, of which 29 were usable.	Audit and feedback by research staff to HVs to increase outcome assessments. Re-contact parents for reminders to complete outcome questionnaires.

For peer review only - http://bmjopen.bmj.com/site/about/guidelines.xhtml

	The number of returned questionnaires was much higher for parents of infants found to be at above population risk.	15 (71%) of parents whose infants were at higher risk returned their follow up questionnaire. Of those at population risk 19 (42%) returned the follow up questionnaire.	
11. Were outcomes measured those that were the most appropriate outcomes?	The proposed primary outcome measure primary outcome measure of weight-for-age z-score.<br>Missing anthropometric data mainly in relation to infant length and head circumference at both baseline and follow-up.<br><br>Low numbers of parents recorded the date of the anthropometric measures on the questionnaires. Of those that did there was a date discrepancy between completion of the self-report aspect of the questionnaires (by parent at home) and the associated anthropometric measures (collected in clinic).<br>The proposed secondary outcomes were parenting self-efficacy, maternal feeding style, infant diet and exposure to physical activity/sedentary behaviour.	Number of infant anthropometric measures completed<br>*Baseline (total N=66)*<br>Weight N=64, Length N=26, Head circumference N=34.<br>*Follow-up (total N=34)*<br>Weight N=28, Length N=15, Head circumference N=14.<br><br>The anthropometric measures on the baseline questionnaire that were dated (N=13) were recorded on average 22 days earlier than the parent report data. The anthropometric measures on the follow-up questionnaire that were dated (N=8) were recorded on average 15 days earlier.<br><br>All follow-up questionnaires that were returned were completed.<br>Cronbach's alpha for the measures was >0.5	Train research team in infant anthropometric measurement and include resources for a follow-up visit by researchers to obviate the need for self-report of infant anthropometrics at baseline and follow-up.<br><br>Ensure anthropometric measurement timing fits with HV service schedule.
12. Was retention to study	Retention was low.	34/66 (52%) of parents completed follow-up	Strategies to maintain participant

For peer review only - http://bmjopen.bmj.com/site/about/guidelines.xhtml

good?		questionnaire. The average time to complete was 28 weeks (min 23, max 39 weeks).<br>Because the intervention was not delivered per protocol there was a time lapse between the assessment and follow-up.<br>12 parents agreed to post-study qualitative interviews.	engagement in study are needed for a larger trial. For example, updates via website, newsletter or text messaging.
13. Were the logistics of running a multicentre trial assessed?	One centre recruited better than the others. This team work well together and there were low levels of staff change and conflict.	Site 1 recruited N=39/109 eligible infants (36%),<br>Site 2 N=15/83 (18%),<br>Site 3 N= 3/10 (30%),<br>Site 4 N= 9/38 (24%).	Training to include team working around peer support, conflict etc.
14. Did all components of the intervention work together?	There were some difficulties blending the ProAsk risk assessment and the motivational approach.<br><br>The Motivational Interviewing (MI) training was too early and insufficient for some HVs.	ProAsk delivered in its entirety to most parents. Only five HVs completed the goal setting stage resulting in a failure to offer follow-up care to parents of infants identified as at above average risk of overweight.<br>Mismatch between MI training and implementation timing.	Communication training for HVs in raising risk and motivational approaches bespoke to childhood overweight prevention.

For peer review only

TABLE 2 DESCRIPTIVE DATA FOR N=53[1] PARTICIPANTS WHO COMPLETED PROASK ASSESSMENT AT BASELINE

Demographic Factors (N=53)	At Population Risk (< 10%)	Above Healthy Risk (≥10%)	**Total**
Gender Boy (%)<br>Girl (%)	 15 (46.9)<br>17 (53.1)	 12 (57.1)<br>9 (42.9)	 **27 (50.8)**<br>**26 (49.1)**
Child age (weeks)	9.84 (1.5)	10.35 (2.3)	**10.04 (1.9)**
Birth weight (kgs)	3.28 (0.5)*	3.86 (0.4)*	**3.51 (0.5)**
Weight for age z-score (WFA-z)	-0.67 (0.6)*	0.32 (0.5)*	**-0.26 (0.7)**
Rapid weight gain (< 0.67 SD)<br>No (%)<br>Yes (%)	 30 (93.8)<br>2 (6.2)	 17 (80.9)<br>4 (19.1)	 **47 (88.7)**<br>**6 (11.3)**
Smoking in pregnancy<br>No (%)<br>Yes (%)	 31 (96.9)<br>1 (3.1)	 21 (100)<br>0 (0)	 **52 (98.1)**<br>**1 (1.9)**
Maternal pre-pregnancy BMI (kg/m^2)<br>**2 missing values*	24.8 (7.2)	27.7 (8.7)	**25.9 (7.9)**
Paternal BMI (kg/m^2)<br>**15 missing values*	26.5 (4.6)*	30.1 (4.2)*	**28.0 (4.8)**
Feeding choice<br>Exclusive breastfeeding (%)<br>Mixed formula and breast (%)<br>Formula only (%)	 11 (34.4)<br>4 (12.5)<br>17 (53.1)	 11 (52.4)<br>2 (9.5)<br>8 (38.1)	 **22 (41.5)**<br>**6 (11.3)**<br>**25 (47.2)**
Mother marital status<br>Married/Living with Partner (%)<br>Single/Separated (%)	 29 (93.6)<br>2 (6.4)	 21 (100)<br>0 (0)	 **50 (96.2)**<br>**2 (3.8)**
Mother employment status<br>Unemployed (%)<br>Part-time (%)<br>Full-time (%)	 3 (9.4)<br>4 (12.5)<br>25 (78.1)	 4 (19.1)<br>5 (23.8)<br>12 (57.1)	 **7 (13.2)**<br>**9 (17.0)**<br>**37 (69.8)**
Education of mother<br>GCSE (%)<br>A Levels (%)<br>Degree (%)<br>Unknown (%)	 12 (37.5)<br>7 (21.9)<br>13 (40.6)<br>0 (0.0)	 8 (38.1)<br>2 (9.5)<br>9 (42.9)<br>2 (9.5)	 **20 (37.7)**<br>**9 (17.0)**<br>**22 (41.5)**<br>**2 (3.8)**
Number of children<br>One (%)<br>Two (%)<br>Three or more (%)<br>Unknown (%)	 11 (34.4)<br>9 (28.1)<br>5 (15.6)<br>7 (21.8)	 7 (33.3)<br>6 (28.6)<br>5 (23.8)<br>3 (14.3)	 **18 (33.9)**<br>**15 (28.3)**<br>**10 (18.9)**<br>**19 (18.9)**
Ethnicity of child<br>White British (%)<br>Non-White British/Mixed/Other (%)	 28 (87.5)<br>4 (12.5)	 19 (90.5)<br>2 (9.5)	 **47 (88.7)**<br>**6 (11.3)**

For peer review only

For peer review only - http://bmjopen.bmj.com/site/about/guidelines.xhtml

ProAsk Risk Score	5.05 (2.3)*	19.4 (8.7)*	**10.7(9.1)**
Income deprivation affecting children(IDACI) ,2015 (N=56)			
Quintile 1 (%)	3 (9)	5 (24)	**8 (15)**
Quintile 2 (%)	4 (13)	6 (29)	**10 (19)**
Quintile 3 (%)	14 (44)	8 (38)	**22 (42)**
Quintile 4 (%)	8 (25)	2 (10)	**10 (19)**
Quintile 5 (%)	3 (9)	0 (0)	**3 (6)**

1. N=56 infants had a ProAsk assessment but three participants had incomplete data transfer from HV to research team.
Categorical variables are numbers and proportions
Continuous variables are means and standard deviations
$*p < 0.05$

For peer review only

TABLE 3A CONTENT ANALYSIS OF QUALITATIVE DATA FOR HEALTH VISITORS (HVS) (N=15)

	No of participants reporting this theme	No of references to this theme
**Eligibility**		
Perception that the study is inappropriate for families where there are maternal mental health concerns or safeguarding issues	8	13
6-8 weeks is a difficult time for HVs to approach parents	8	14
Language barriers problematic	9	18
**Recruitment**		
HV participated at manager's request	9	15
HV workload made it difficult to prioritise study	5	6
Wariness about raising the study with some parents because of expectations that they might be offended or overloaded	6	6
HV engagement with project was supported by positive professional relationships	4	11
Belief that more educated parents were more interested in the study	6	9
**Consent**		
Belief that some parents need a relationship with a professional before they will engage/permit home access	6	11
Belief that parental wariness of unknown researcher negatively impacted on the numbers of participants giving written consent	4	7
**Adherence to intervention**		
Difficulties feeding back overweight risk score from programme to parents	4	8
Therapeutic wheel used with all parents	3	5
All elements of the wheel discussed	7	12
**Intervention Acceptability**		
Concern about unintended consequences of overweight risk identification	8	14
Belief that early prevention is better than later management	9	13
Belief that timing of ProAsk personalised risk communication for infants is too early for parents	9	17
ProAsk is engaging to use	7	18
ProAsk wheel (on tablet) contained useful information	7	9
ProAsk tablet a disappointment	1	1
3-4 months is an appropriate time for this intervention	3	3
**Components of protocol working together**		
Belief that HVs already do this work	7	10

For peer review only

For peer review only - http://bmjopen.bmj.com/site/about/guidelines.xhtml

Training day should have been closer to study start date	7	9
Lack of tablet device during training a problem	5	7
Initial lack of confidence in explaining study to potential participants	4	6
HV misunderstood study protocol	5	6
ProAsk on tablet a challenge for novel users	8	18
Belief that ProAsk risk assessment should be accompanied by intervention and input by HV	6	21
Team work and practice improved skills in using tablet	6	15

For peer review only

For peer review only - http://bmjopen.bmj.com/site/about/guidelines.xhtml

TABLE 3B CONTENT ANALYSIS OF QUALITATIVE DATA FOR PARENTS (N=12)

	No of participants reporting this theme	No of references to this theme
**Recruitment**		
Parent felt study recruitment processes acceptable	11	13
Parent felt well informed about the study	10	11
Parent participated because of own weight issues/issues with family eating patterns	7	12
Parent participated for altruistic reasons	7	12
Parent willing to be randomised to participate in future trial.	11	11
**Adherence to intervention**		
No recall of, or uncertainty about, feedback of personalised overweight risk score for baby	4	7
Raised awareness/change in perception in response to overweight risk feedback and intervention	3	3
Parent reports no behaviour change following ProAsk	4	5
**Intervention Acceptability**		
Parental belief that they are already doing the right thing	6	9
Belief that early prevention is better than cure	3	5
Receiving risk score was upsetting	1	1
Receiving risk score was a relief	2	2
Parent would have liked more e.g. app, website, on-going information	3	5
**Outcome assessment**		
Questionnaire components a cue to behaviour change	4	5
**Components of protocol working together**		

For peer review only

For peer review only - http://bmjopen.bmj.com/site/about/guidelines.xhtml

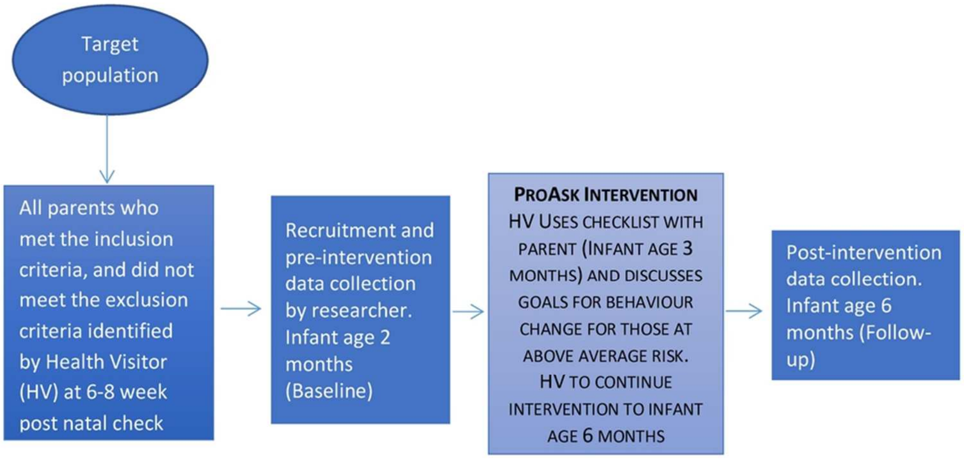

Study Regimen

84x40mm (300 x 300 DPI)

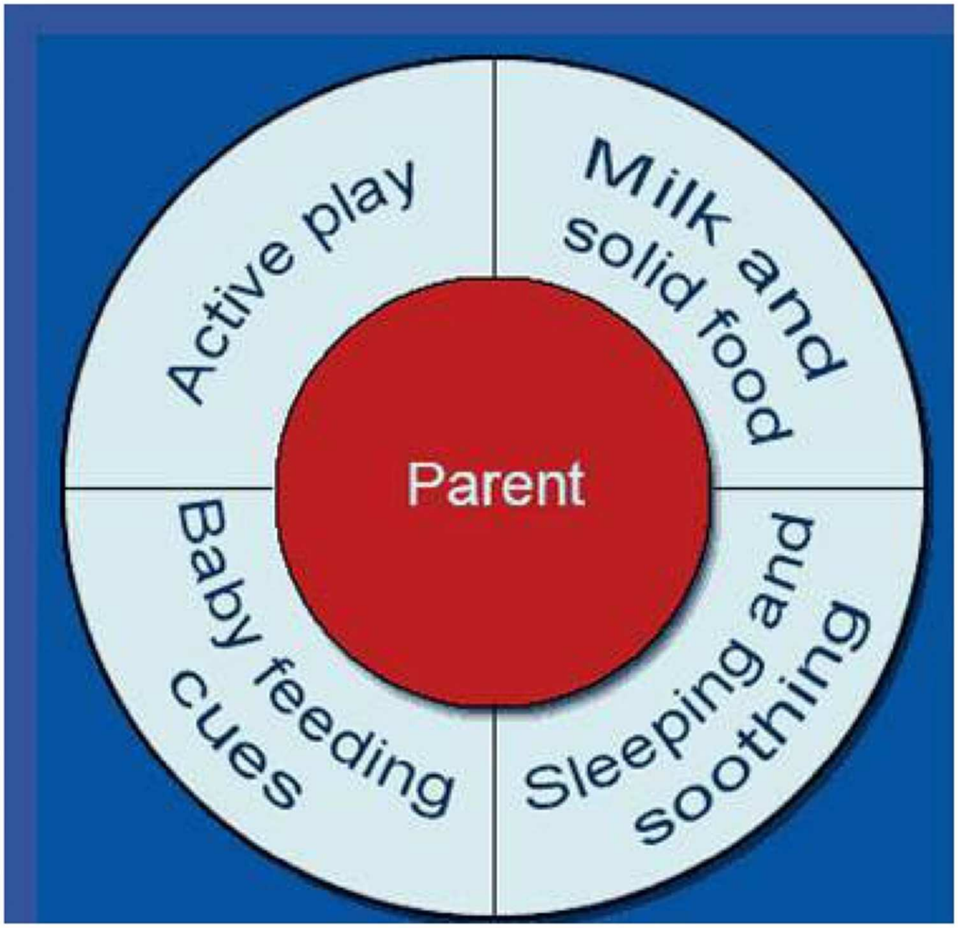

Therapeutic wheel showing the options to support healthy weight

72x70mm (300 x 300 DPI)

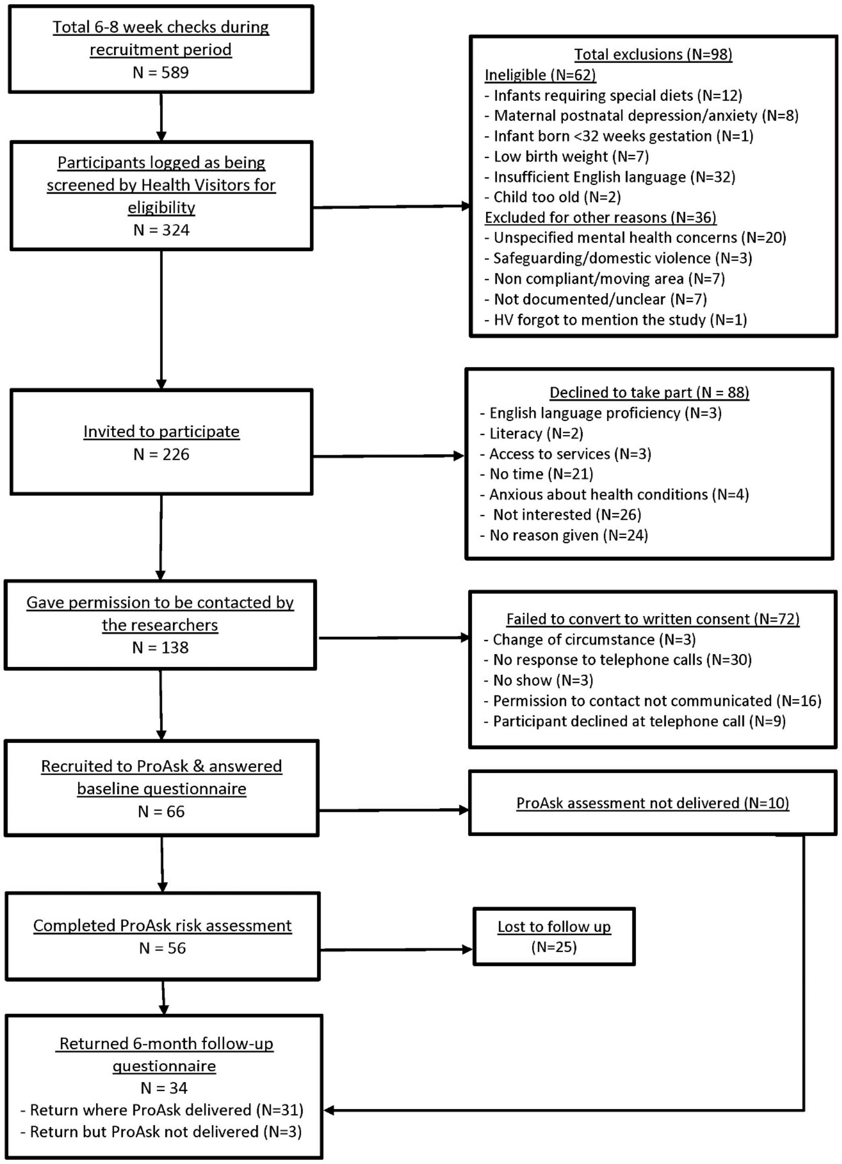

Study Recruitment Flow Chart

183x253mm (300 x 300 DPI)

SUPPLEMENTARY FILE 1 - SUMMARY OF DATA COLLECTION MEASURES

Methodological issue and associated study objective	Quantitative measures	Qualitative data collected
1. Did the feasibility study allow a sample size calculation for the main trial?	Researchers recorded the number of infants recruited.<br>Parent self-report of infant weight and age at baseline and 6 months enabled changes in weight for age z-scores for in infants at average population risk and above population risk of overweight to be calculated.	N/A
2. What factors influenced eligibility and what proportion of those approached were eligible?	The NHS Child Health Records were examined to determine the number of infants that received their 6-8 week check in the respective study sites during the recruitment period.<br>Health visitor (HV) log sheets recorded the number of 6-8 week old infants receiving home visits, the number of parents who were eligible for the study and were approached about participating, the number of parents who declined and their reasons (where given) for declining.	Interviews with HVs to explore practitioners' experiences of recruiting to the feasibility study.
3. Was recruitment successful?	Comparison of expected and observed recruitment.<br>Recruitment rate anticipated to be N=100 in three months (based on data provided by NIHR-CRN co-ordinator). Any extension to the recruitment period.	Interviews with HVs to explore their perspective on the recruitment processes.

For peer review only

4. Did eligible participants consent?	HV records of numbers of eligible infants and numbers of parents who agreed to be approached by the researcher. Researcher records of parents successfully recruited.	Interviews with HVs to explore their perspective on the recruitment processes.
5. Were participants successfully randomised and did randomisation yield equality in groups?	Not assessed	Not assessed
6. Were blinding procedures adequate?	Not assessed	Not assessed
7. Did participants adhere to the intervention?	Proportion of participants who received the ProAsk assessment and intervention for those at greater risk.	Interviews with HVs explored intervention adherence. Cross referencing of data on overweight risk downloaded from the ProAsk tablets with interview data from parents allowed researchers to investigate key elements of intervention adherence.
8. Was the intervention acceptable to the participants?	Numbers of parents and HVs who took part in the study	Interviews with HVs and parents explored their views on the acceptability of the intervention.
9. Was it possible to calculate intervention costs and duration?	Not assessed	Not assessed
10. Were outcome assessments completed	Completion rates for risk assessment, baseline and 6 month follow-up questionnaires.	
11. Were outcomes measured those that were the most appropriate outcomes?	Proportion and type of missing data in ProAsk risk assessment and follow-up measures	Interviews with parents exploring their views of study processes.
12. Was retention to study good?	Retention rate - from consent to follow-up at 6 months.	
13. Were the logistics of running a multicentre trial assessed?	Recruitment and intervention completion rates for all four sites.	Interviews with HVs probed their views of the study processes.

For peer review only

14. Did all components of the intervention work together?	Completion rates of ProAsk assessment.	Interviews with HVs and parents explored the interviewees' experiences of intervention components, including IROC assessment, risk feedback, therapeutic wheel, motivational interviewing techniques, goal setting and follow-up.

For peer review only
